# Exploring the Contribution of Autophagy to the Excess-Sucrose Response in *Arabidopsis thaliana*

**DOI:** 10.3390/ijms23073891

**Published:** 2022-03-31

**Authors:** Daniel Laloum, Sahar Magen, Yoram Soroka, Tamar Avin-Wittenberg

**Affiliations:** Department of Plant and Environmental Sciences, Alexander Silberman Institute of Life Sciences, The Hebrew University of Jerusalem, Jerusalem 9190401, Israel; daniel.laloum@mail.huji.ac.il (D.L.); sahar.magen@mail.huji.ac.il (S.M.); yorams@ekmd.huji.ac.il (Y.S.)

**Keywords:** *Arabidopsis thaliana*, autophagy, sucrose, mitochondria, peroxisome, reactive oxygen species (ROS)

## Abstract

Autophagy is an essential intracellular eukaryotic recycling mechanism, functioning in, among others, carbon starvation. Surprisingly, although autophagy-deficient plants (*atg* mutants) are hypersensitive to carbon starvation, metabolic analysis revealed that they accumulate sugars under such conditions. In plants, sugars serve as both an energy source and as signaling molecules, affecting many developmental processes, including root and shoot formation. We thus set out to understand the interplay between autophagy and sucrose excess, comparing wild-type and *atg* mutant seedlings. The presented work showed that autophagy contributes to primary root elongation arrest under conditions of exogenous sucrose and glucose excess but not during fructose or mannitol treatment. Minor or no alterations in starch and primary metabolites were observed between *atg* mutants and wild-type plants, indicating that the sucrose response relates to its signaling and not its metabolic role. Extensive proteomic analysis of roots performed to further understand the mechanism found an accumulation of proteins essential for ROS reduction and auxin maintenance, which are necessary for root elongation, in *atg* plants under sucrose excess. The analysis also suggested mitochondrial and peroxisomal involvement in the autophagy-mediated sucrose response. This research increases our knowledge of the complex interplay between autophagy and sugar signaling in plants.

## 1. Introduction

Macroautophagy (hereafter termed autophagy) is a molecular mechanism common to all eukaryotes, involved in the degradation and recycling of cellular components ranging from single proteins to entire organelles. It functions in the elimination of various proteins under stress and favorable conditions. In plants, autophagy was found to be essential for many stress responses, especially stresses involving nutrient deficiency [1,2,3]. Under carbohydrate starvation, autophagy was found to be associated with the recycling of proteins, amino acids, and lipids as alternative energy sources [4,5,6,7,8,9]. Plants with impaired autophagic activity (*atg* mutants) are hypersensitive to carbon starvation. Under such conditions, they display a variety of metabolic alterations, including sugar accumulation [9,10].

Sucrose is a primary carbohydrate that can be produced solely by oxygenic photosynthetic organisms. In plants, sucrose serves both as a nutrient and as a signaling molecule essential for many physiological and developmental processes [11]. Since sucrose is comprised of both fructose and glucose, many responses linked to glucose are also associated with sucrose [12]. In the shoot, excess sucrose and glucose lead to the accumulation of anthocyanins [13,14]. In the root, low concentrations of exogenous sucrose or glucose promote root formation, while higher concentrations inhibit root growth [15,16,17].

Several mechanisms found to mediate the glucose response were also observed in response to sucrose and can be categorized into the hexokinase-dependent and -independent pathways [18,19]. The sucrose and glucose excess response also involves many phytohormones such as abscisic acid (ABA), auxin, and cytokinin [20,21,22,23,24,25]. While many unique sucrose responses have been observed, the sensing mechanism is difficult to define since sucrose is hydrolyzed into hexoses such as glucose, fructose, and trehalose-6-phosphate (T6P) at an early stage [11,26]. Recent studies found that the sucrose response is mediated by various mechanisms involving transcription, translation, post-translation, protein interaction, and hormone-related processes [26].

T6P levels change in accordance with sucrose levels [27,28,29] and play a major role in plant development. Mutations in trehalose phosphate synthase lead to delayed embryo development [30]. It was also found that T6P inhibits SNF 1-related protein kinase 1 (SnRK1 [31,32,33]), which is a regulator of many stress responses and energy metabolism [33,34,35,36]. Sucrose is also hydrolyzed into fructose, but fructose signaling differs from glucose and relies on other genes and transporters [37,38]. Moreover, in contrast to glucose and sucrose, exogenous fructose inhibits root growth at early stages [17,37,38].

A recent study showed that autophagy plays a role in glucose-mediated inhibition of root formation by promoting peroxisomal degradation under conditions of reactive oxygen species (ROS) accumulation. The authors observed that an absence of autophagy led to the accumulation of peroxisomes, resulting in lower ROS levels and higher levels of bioavailable auxin, mediated via ABC transporter 1 (ABCD1) activity [39]. These findings align with previous works that characterized the selective degradation of peroxisomes by autophagy [40,41,42]. Resonating with these observations, we reported on altered peroxisomal activity in *atg* mutants under dark-induced senescence [6].

The current research set out to investigate the role of autophagy under conditions of excessive exogenous sucrose, comparing wild-type (WT) and *atg* mutant plants exposed to these conditions. We demonstrate that autophagy plays a role in the root stress response under excess sucrose and present an extensive proteomic analysis revealing candidate mediators of the sucrose response affected by the autophagy mechanism. Surprisingly, it seems that the effect of excess sucrose in *atg* mutants is mostly signaling-related rather than metabolic. We found that under excess sucrose, *atg* mutant plants had elevated levels of proteins related to ROS detoxification, alleviating root shortening. These results refine the previously reported model of the role of autophagy during sugar excess and expand our knowledge regarding the interplay between autophagy and sugar signaling in plants.

## 2. Results

### 2.1. atg Mutants Display Reduced Sensitivity to Sucrose Excess

**Figure 1:**
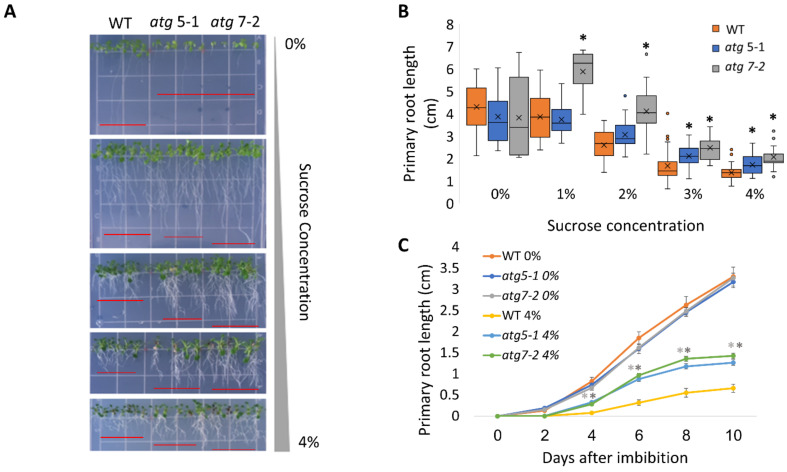
*atg* mutants display reduced sensitivity to sucrose excess. (**A**,**B**). WT, *atg5-1*, and *atg7-2* plants were sown on Nitsch plates containing increasing concentrations of sucrose, and grown vertically for 14 days. (**A**) Representative image of the plants. (**B**) Quantification of primary root length under various sucrose treatments. Asterisk denotes a significant difference from WT under the same treatment conditions, as determined by Dunnett’s test (*p* < 0.05, *n* = 10–30), x indicates sample mean, and circles denote outliers. (**C**) WT, *atg5-1*, and *atg7-2* plants were sown on Nitsch plates containing 0% or 4% sucrose and grown vertically. The primary root length was measured every two days for 10 days after imbibition. Data are presented as the average ± SE. An asterisk denotes a significant difference from WT under the same treatment conditions and at the same time point, as determined by Dunnett’s test (*p* < 0.05, *n* = 12–31). Dark grey—*atg5-1*, light grey—*atg7-2*.

**Figure 2:**
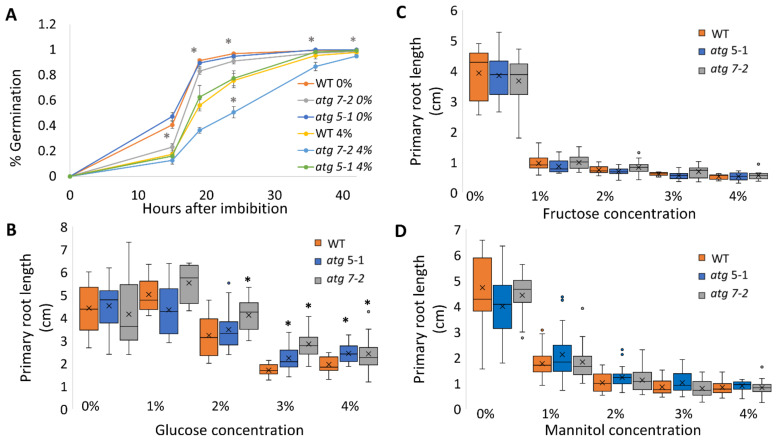
Less root length inhibition in *atg* mutants under sucrose and glucose but not fructose. WT, *atg5-1*, and *atg7-2* plants were sown on Nitsch plates containing increasing concentrations of sugars (molar equivalent to sucrose concentration). (**A**) Germination percentage (GP) of WT, *atg5-1*, and *atg7-2* seeds under 0% and 4% sucrose. Data are presented as the average ± SE. An asterisk denotes a significant difference from WT under the same treatment conditions and at the same time point, as determined by Dunnett’s test (*p* < 0.05, *n* = 4–6); light grey—*atg5-1*, dark grey—*atg7-2*. (**B**) Quantification of primary root length under various glucose treatment conditions. An asterisk denotes a significant difference from WT under the same treatment by Dunnett’s test (*p* < 0.05, *n* = 8–24). x indicates sample mean, and circles denote outliers. (**C**) Quantification of primary root length under various fructose concentrations. No significant difference from WT under the same treatment condition was found by Dunnett’s test (*p* < 0.05, *n* = 9–17). x indicates sample mean, and circles denote outliers. (**D**) Quantification of primary root length under various mannitol treatments. No significant difference from WT under the same treatment condition was found by Dunnett’s test (*p* < 0.05, *n* = 16–22). x indicates sample mean, and circles denote outliers.

To assess the role of autophagy in the cell response to excess sugar, we set out to explore the behavior of *atg5-1* and *atg7-2*, two previously described autophagy mutants [43], under various sucrose concentrations. ATG5 and ATG7 both participate in ATG8 lipidation during autophagosome membrane expansion, and their T-DNA insertion lines are extensively used to study autophagy in plants [44,45,46]. Plants were sown on Nitsch plates containing different sucrose concentrations (0–4%), and the seedling morphology was examined 14 days post-imbibition. As previously reported for WT plants, we observed anthocyanin accumulation in the shoots in response to high sucrose concentrations (Figure 1A and Figure S1A [13,14]). No phenotypic differences were observed between the shoots of WT and the *atg* mutant plants. In addition, we observed significant root shortening in response to sucrose excess, as previously described [17]. Interestingly, *atg* mutants displayed reduced sensitivity, as reflected by a smaller effect of sucrose excess on their root system compared to WT, while no visible differences were observed in lateral roots between the lines (Figure 1A,B). To further characterize the exact time the difference in root length manifested in *atg* mutants under sucrose excess, primary root growth was monitored over time. Under no sucrose, the roots grew to the same extent and at the same rate as WT roots. However, under sucrose excess conditions, significant differences in the primary root length were observed as early as 2–4 days post-imbibition (Figure 1C and Figure S1B–D). To ascertain whether the differences in root length stemmed from delayed seed germination, the seed germination of WT and *atg* mutant plants was monitored under increasing sucrose concentrations. Although sucrose excess reduced the overall seed germination rates, WT and *atg5-1* seeds germinated at similar rates and were both faster than *atg7-2* (Figure 2A and Figure S2). These results demonstrate that the root length phenotype observed for *atg* mutants under sucrose excess is directly connected to root growth and was not a result of altered seed germination.

### 2.2. Less Root Length Inhibition in atg Mutants under Sucrose and Glucose but Not Fructose Excess

As sucrose is a disaccharide comprised of glucose and fructose, the altered root response of *atg* mutants to sucrose, glucose, and fructose treatments was then assessed. To this end, the experiment described above was repeated with glucose and fructose instead of sucrose, at molar concentrations of glucose and fructose accounting for the conversion from a disaccharide to monosaccharides. When grown under increasing glucose concentrations, a trend similar to that observed with sucrose was observed (Figure 2B and Figure S3A), as previously described [39]. In contrast, when grown in the presence of fructose, all plant roots were shortened to the same extent irrespective of their genotype, and the stress response was visible at a relatively low concentration (Figure 2C and Figure S3B). To further confirm that the response is unique to sucrose and glucose and not due to general osmotic stress, the experiments were repeated in plates containing increasing concentrations of mannitol corresponding to sucrose molar concentrations. In this case, all plant lines exhibited a similar reduction in root length. However, the phenotype was not as severe as that observed under fructose treatment (Figure 2D and Figure S3C).

### 2.3. Sucrose Excess Induces Autophagy in the Shoots but Not in Roots

**Figure 3:**
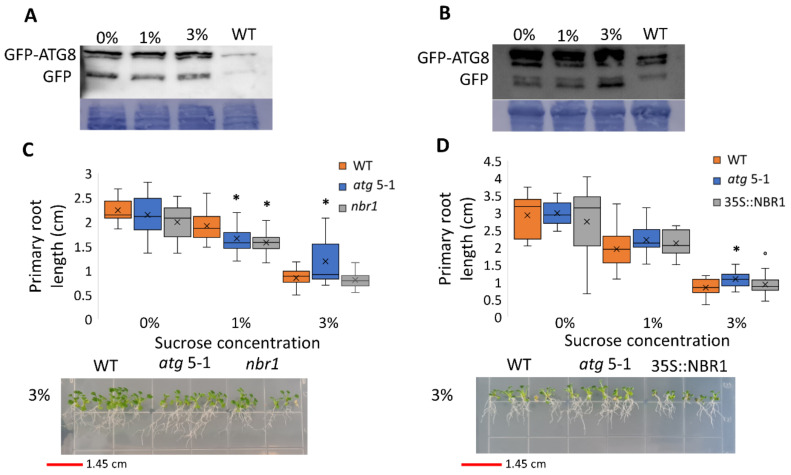
Sucrose excess induces autophagy in the shoots but not in the roots, and the root phenotype is not NBR1-dependent. (**A**,**B**) GFP-ATG8f plants were sown on Nitsch plates containing increasing concentrations of sucrose, and grown for 10 days. (**A**) Roots and (**B**) shoots were collected separately, and proteins were extracted and analyzed by Western blot using an anti-GFP antibody. Equal protein loading was verified by Coomassie brilliant blue staining. (**C**) WT, *atg5-1*, *nbr1*, or (**D**) NBR-OX (35S::NBR1) plants were sown on Nitsch plates containing increasing concentrations of sucrose and grown vertically for 14 days, after which the primary root length was quantified (top panel). An asterisk denotes a significant difference from WT under the same treatment, as determined by Dunnett’s test (*p* < 0.05, *n* = 10–30), x indicates sample mean, and circles denote outliers. Bottom panel—a representative image of the plants.

Since *atg* mutants displayed reduced root but not shoot sensitivity to sucrose excess, the possibility of differential autophagy induction in these tissues under sucrose excess was examined. To this end, autophagy activity was measured using the GFP-ATG8 release assay [47]. ATG8 is a protein located at the membrane of the autophagosome [48]. Following autophagosome fusion with the vacuole and its subsequent degradation, the GFP-ATG8 fusion protein is degraded, although free GFP is more stable than the fusion protein. The GFP-ATG8 fusion protein can be followed by Western blot analysis and the release of free GFP is indicative of autophagosome fusion with the vacuole. Roots and shoots of 10-day-old GFP-ATG8f plants were grown on various sucrose concentrations and GFP release was examined by Western blot. Analysis of proteins from root samples revealed no changes in free GFP levels, indicating no differences in autophagic activity (Figure 3A and Figure S4A) and suggesting that the autophagic activity responsible for altered root development under sucrose excess was basal autophagy. Interestingly, under excess sucrose conditions, an induction of autophagic activity was observed in shoot samples (Figure 3B and Figure S4B). This induction was not reflected by a morphological phenotype of WT compared to *atg* mutant shoots.

Neighbor of BRCA1 (NBR1) is a selective autophagy cargo receptor found to be essential for the autophagy response under various abiotic stresses [49] and to modulate ABA signaling [50]. To determine whether NBR1 is involved in the root response to excess sucrose, the responses of an NBR1 knockout (KO) line (*nbr*1) and an NBR1 overexpression line (35S::NBR1) [50] to sucrose excess were characterized. Unlike *atg5-1* and *atg7-2*, the primary root length of both *nbr1* and 35S::NBR1 was similar to that of WT under high sucrose concentrations (Figure 3C,D), demonstrating that the response was not NBR1-dependent. Taken together with the GFP-release observations, we postulated that the reduced root sensitivity of *atg* mutant plants under sucrose excess is dependent on bulk rather than selective autophagy.

### 2.4. No Difference in Starch Accumulation between WT and atg Mutants under Sucrose Excess

**Figure 4:**
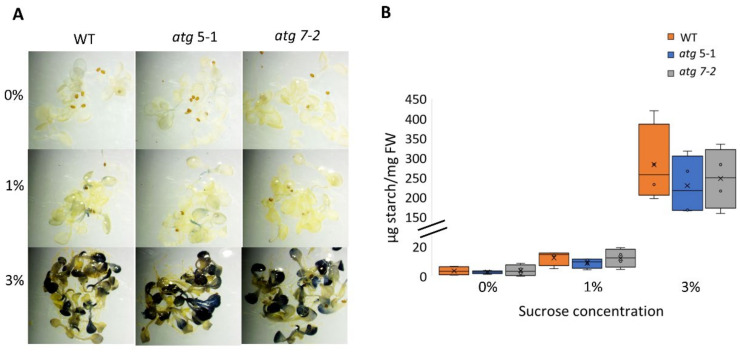
No difference in starch accumulation between WT and *atg* mutants under sucrose excess. WT, *atg5-1*, and *atg7-2* plants were sown on Nitsch plates containing increasing concentrations of sucrose and grown for 14 days. (**A**) Representative images of IKI staining of starch. (**B**) Quantification of starch. No significant difference from WT under the same treatment was found by Dunnett’s test (*p* < 0.05, *n* = 4), x indicates sample mean, and circles denote outliers.

Several studies have demonstrated that *atg* mutants display altered metabolism under both favorable and stress conditions [6,10,51,52,53,54]. It was thus postulated that the reduced sensitivity of *atg* mutant roots to sucrose excess might stem from differential usage of the sucrose available in the medium and its transport to shoots. As sucrose can be used as a substrate for starch synthesis [55], the predominant mode of carbohydrate storage in plants, the starch levels in WT and *atg* mutant plants exposed to increasing sucrose levels were quantified (Figure 4). Lugol’s potassium iodide (IKI) staining and starch quantification showed that elevated sucrose levels led to higher starch concentrations in the leaves, but not to differences between lines under the same treatment (Figure 4A,B). These findings indicate that the sucrose available in the medium was not differentially utilized in the different plant lines. The root response of *atg* seedlings might be a result of sucrose signaling rather than its role as a metabolite.

### 2.5. Sucrose Excess Affects the Plant Metabolome in an Autophagy-Independent Manner

**Figure 5:**
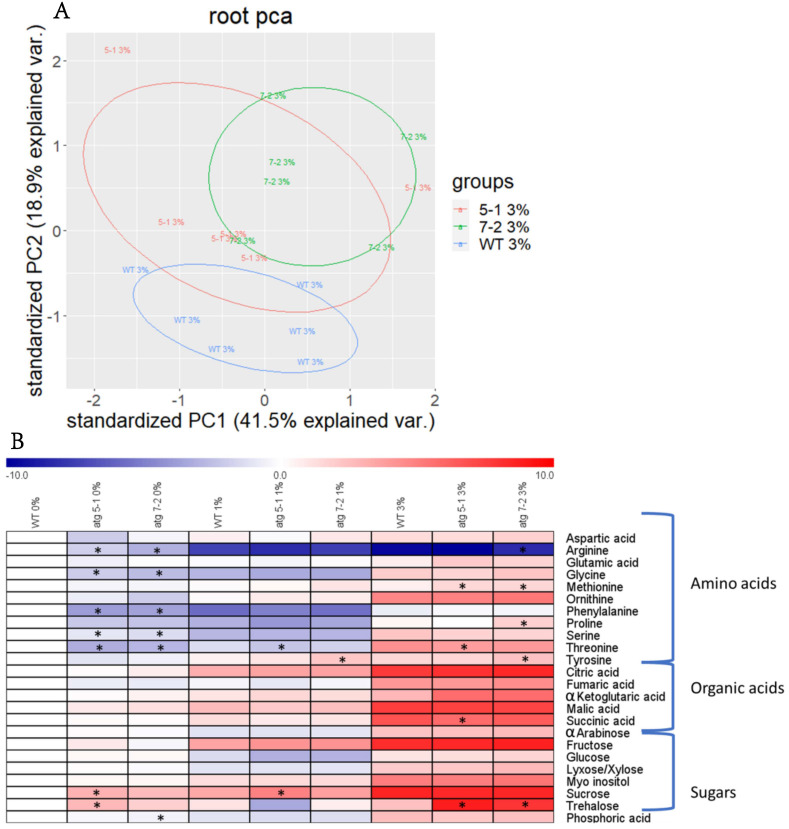
Sucrose excess affects the root metabolome, but the influence of autophagy on the response is mild. Roots from WT, *atg5-1*, and *atg7-2* seedlings grown for 10 days under various sucrose concentrations were collected, and polar metabolites were extracted and analyzed by gas-chromatography mass-spectrometry (GC-MS) (**A**). Principal component analysis (PCA) performed on scaled values of metabolites measured in roots grown under 3% sucrose (**B**). Heatmap displaying the relative amounts of root metabolites under various sucrose concentrations. Results are presented as the log2 ratio normalized to WT under 0% sucrose. An asterisk denotes a significant difference from WT under the same treatment regimen, as determined by Dunnett’s test (*p* < 0.05, *n* = 5–6).

Due to the substantial effect previously shown of autophagy on plant metabolism [5,6,8,9,10], the metabolic profile of WT and *atg* mutant roots and shoots from plants grown under sucrose excess was evaluated. After growing seedlings for 10 days, as described above, polar metabolites were extracted and analyzed by gas-chromatography mass-spectrometry (GC-MS). Shoot analysis revealed distinct metabolite profiles for each treatment, but no differences between the lines (Figure S5A, Table S1). Principal component analysis (PCA) of root metabolites demonstrated that, as in the shoots, sucrose excess had a significant effect on metabolic profiles (Figure S5B). In addition, when performing PCA of each individual treatment, minor differences between the lines were noted (Figure 5A and Figure S5C,D).

Heat map visualization of the roots revealed that under no sucrose, *atg* mutants displayed lower levels of amino acids and higher levels of sugars compared to WT (Figure 5B). These results matched the profile previously described for plants following carbon starvation [10]. Elevation of sugar levels dramatically increased the levels of most observed metabolites in both WT and *atg* mutants, but the differences between the lines became milder (Figure 5B, Table 1 and Table S2). Trehalose (Figure 5B, Table 1), a sugar previously found to mediate the oxidative stress response [56], was shown to accumulate under sucrose excess in *atg* mutants. Taken together with the starch accumulation results, it can be concluded that the altered response of *atg* mutants to sucrose excess likely stems from sucrose functioning as a signaling factor rather than as a metabolic agent.

### 2.6. Differences in Protein Levels under Sucrose Excess and between WT and atg5-1 Plants

In light of the findings suggesting that sucrose serves as a signaling molecule in root responses to sucrose excess and due to the role of autophagy in protein elimination, it was postulated that the protein profiles of WT and *atg* mutant roots will differ under such stress conditions [57,58,59]. To assess this possibility, WT and *atg5-1* plants were exposed to 1% (non-excess conditions) and 3% sucrose (excess conditions), and the protein profiles of the roots were analyzed by liquid chromatography with tandem mass spectrometry (LC-MS/MS).

**Figure 6:**
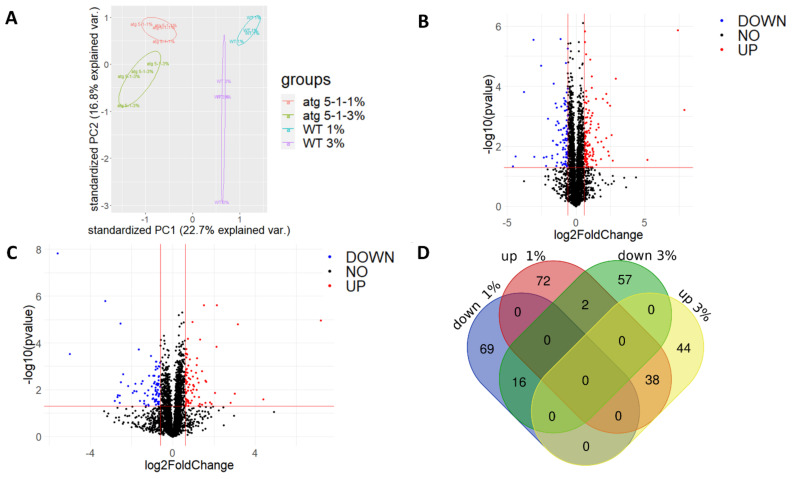
Proteomic analysis of roots revealed differences in protein levels during sucrose excess and between WT and *atg5-1* plants. WT and *atg5-1* were sown on Nitsch plates containing 1% or 3% sucrose and grown vertically for 10 days. Roots were then collected and subjected to proteomic analysis by liquid chromatography with tandem mass spectrometry (LC-MS/MS). (**A**) PCA of the label-free quantification (LFQ) values of the entire proteome. (**B**) Volcano plot for 1% sucrose—*p* values and fold-change relative to *atg5-1*. (**C**) Volcano plot for 3% sucrose—*p* values and fold-change relative to *atg5-1*. (**D**) Venn diagram of differentially accumulated proteins: http://bioinformatics.psb.ugent.be/webtools/Venn/, accessed on 26 December 2021.

PCA revealed that while a clear separation was observed between the lines and sucrose treatments, it was more significant between the lines as compared with the treatment (Figure 6A). Similar trends were observed for the clustering of the calculated Z scores of protein levels between lines vs. across treatments (Figure S6A). Taken together, these results indicate that although a sucrose excess affects the cell proteome, the lack of autophagy also plays a significant role in determining the root proteome under both normal and excess sucrose conditions.

A comparison of the proteome profiles found that many proteins differentially accumulated both under 1% sucrose and 3% sucrose (Figure 6B,C). A Venn diagram drawn to identify proteins that only changed under 3% sucrose revealed 57 proteins that were downregulated and 44 that were upregulated in *atg5-1* under the 3% and not the 1% sucrose (Figure 6D).

### 2.7. Lack of Autophagy Results in Alterations to a Plethora of Biological Processes under High Sucrose Levels

**Figure 7:**
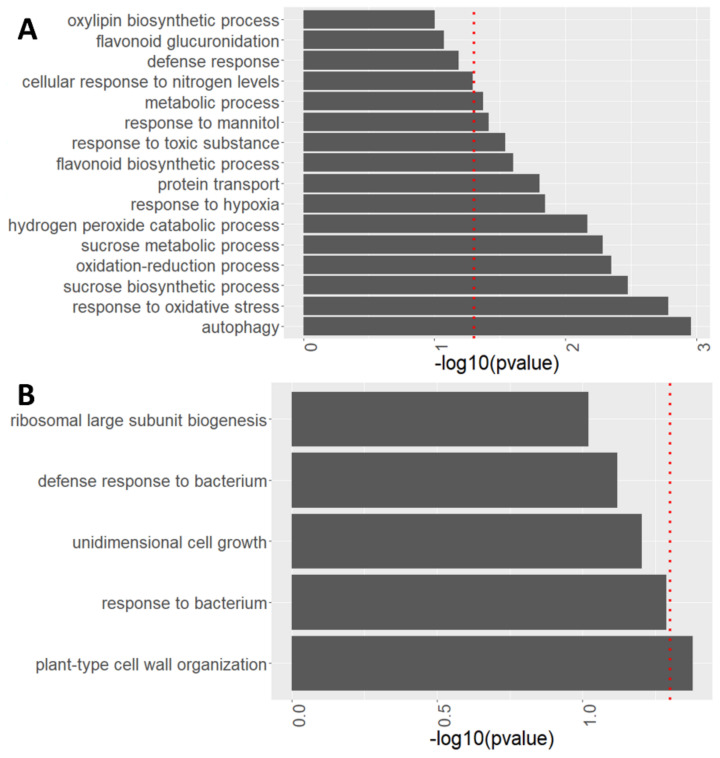
*atg5-1* shows alterations in many biological processes under high sucrose levels. Enriched biological processes identified from proteomics analyses performed using David functional annotation tools in GOTERM_BP_DIRECT. (**A**) Biological processes involving protein accumulation in *atg5-1* compared to WT under 3% sucrose. (**B**) Biological processes involving protein depletion in *atg5-1* compared to WT under 3% sucrose. The red dotted line indicates the threshold for significant enrichment of the given proteins for the biological process (*p* < 0.05).

After observing global changes in protein abundance in *atg* roots compared to WT, the specific processes modulated by autophagy with and without sucrose excess treatment were characterized via biological process enrichment analysis performed using David functional annotation tools (V6.8). Autophagy was one of the most affected processes, upregulated in *atg5-1* mutants both under 1% and 3% sucrose (Figure 7A and Figure S5).

Several biological processes were upregulated in *atg5-1* mutant roots under the 3% sucrose treatment (Figure 7A). Upregulation of a hydrogen peroxide catabolic process was observed under sucrose excess treatment. This involved increased levels of peroxidase 15 (PER 15), peroxidase 23 (PER23), peroxidase 51 (PER51), and cationic amino acid transporter 2 (CAT2) (Figure 7, Table 2). In addition, increased levels of three IAA-amino acid hydrolases of the ilr1-like family proteins—ILL1, 2 and —were observed (Table 2). These proteins were found to hydrolyze amino acids from auxin, leading to the accumulation of an active form of auxin [60]. These processes were also significantly upregulated under 1% sucrose (Table 2), suggesting that the increased levels of the proteins might serve as a pre-existing condition, aiding in the plant response to sucrose excess in combination with processes specifically activated under these conditions.

One protein found to accumulate to a great extent in *atg5-1* under sucrose excess was uncoupler protein 5 (PUMP5) (Table 2). In parallel, an accumulation of TCA cycle proteins in *atg5-1* under sucrose excess was observed (Table 2). These included citrate synthase, and succinate dehydrogenase subunit 7, which significantly accumulated under sucrose excess in *atg5-1* plants, and succinyl CoA ligase, which showed higher (but not significant) levels. Since uncoupler proteins can finetune the mitochondrial membrane potential, and consequently lead to a reduction in ROS production [61,62,63,64], these findings strongly suggest mitochondrial involvement in the root response to excess sucrose.

In parallel, fructose biphosphate aldolase 4 (FBA4) exhibited a 237.2-fold upregulation under 1% sucrose (Supplementary Data S3). FBAs are key metabolic enzymes catalyzing the reversible cleavage of fructose 1,6 biphosphate into dihydroxyacetone phosphate and glyceraldehyde-3-phosphate. This reaction occurs during glycolysis, gluconeogenesis, and the TCA cycle [65,66]. Under 3% sucrose, however, FBA4 accumulated 30.3 fold in *atg5-1* compared to WT, but the difference was not statistically significant (Supplementary Data S2). These results imply a strong involvement of autophagy in FBA4 regulation, independent of sucrose excess. Sucrose metabolism-related proteins, comprising sucrose synthase 1, 3, and 4, also accumulated in *atg5-1* plants (Figure 7A, Table 2). These enzymes are the most abundant sucrose synthases in the root system, and knockout plants of these genes were found to be highly sensitive to hypoxia [67]. Upregulation of these proteins was also observed under 1% sucrose but not as severely as in stress conditions. Sucrose synthases are mostly associated with the degradation of sucrose and the accumulation of glucose and fructose [67,68]. Yet, since these reactions are reversible, it is difficult to determine the exact role of sucrose synthase, but it might explain previous results demonstrating sugar accumulation in *atg* mutants. [10,39].

When studying downregulated processes, various cell-wall responses were observed, which involved two common proteins: expansin A1 (EXPA1) and expansin 11 (EXPA11). Both proteins are essential for cell-wall expansion (Figure 7B, Table 3) and cell enlargement and were found to be necessary for plant growth, development, and the response to stress [69,70,71]. It has been previously reported that *rns*2-2 plants, which lack the T2 ribonuclease RNS2, display enhanced basal autophagy. The mutants were larger than WT plants, and the authors postulated this was due to higher expansin levels [72]. The current results suggest that autophagy is involved in expansin maintenance. However, since there is a discrepancy between the phenotypes of *rns2-2* and *atg* mutants, more studies are needed to determine the nature of this regulation.

In addition to the accumulation of auxin-related response proteins essential for auxin bioavailability, changes in auxin transport proteins, e.g., decreased PIN LIKE 5 (PILS5), were observed in *atg5-1* roots under 3% sucrose (Table 3). PILS5 is an auxin carrier that controls auxin sequestration in the ER, leading to lower cytosolic auxin levels. This affects both auxin availability and signaling [73,74].

### 2.8. atg Mutants Display Higher Peroxidase Activity under Sucrose Excess Compared with WT

**Figure 8:**
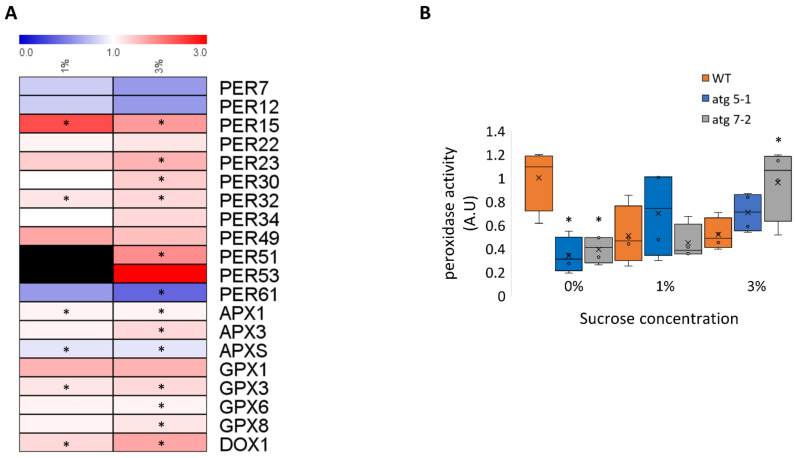
Root peroxidase activity is increased in *atg* mutant plants under sucrose excess (**A**). Heatmap describing the fold-change in peroxidase levels between *atg5-1* and WT under 1% and 3% sucrose. Black boxes indicate peroxidases that were not detected. An asterisk denotes a significant change between WT and *atg5-1*, as determined by Student’s *t*-test. (**B**). WT, *atg5-1*, and *atg7-2* plants were sown on Nitsch plates containing 0%, 1%, or 3% sucrose and grown vertically for 10 days. Roots were collected and the peroxidase activity was assessed. An asterisk denotes a significant difference from WT under the same treatment, as determined by Dunnett’s test (*p* < 0.05, *n* = 4), x indicates sample mean, and circles denote outliers.

In light of the observed accumulation of proteins related to hydrogen peroxide catabolism in *atg5-1* mutants, we wished to determine whether these affected their impact on hydrogen peroxide degradation. Most of the peroxidases identified in the proteomic analysis accumulated in *atg5-1* plants under 1% and 3% sucrose, with a slightly higher accumulation under sucrose excess (Figure 8A, Supplementary Data S2). Analysis with Uniprot [75] revealed that these peroxidases were mostly extracellular. Quantification of peroxidase activity in roots of 10-day-old seedlings revealed that under 0% sucrose, WT roots displayed higher peroxidase activity than *atg* mutants. In contrast, under 1% sucrose, no differences in peroxidase activity were detected between the lines, while under 3% sucrose, *atg* mutants display higher peroxidase activity than WT plants (Figure 8B). These results further confirmed the involvement of autophagy in ROS homeostasis under sucrose excess, likely stemming from the accumulation of peroxidases.

## 3. Discussion

The hypersensitivity of *atg* mutant plants to carbon starvation is a well-defined phenotype [5,76]. Surprisingly, it has been shown that under such conditions, *atg* mutants accumulate higher levels of sugars, which is counter-intuitive, considering their apparent increased starvation [9,10]. It is also known that autophagy is activated according to the sugar status of the plant [77,78], suggesting a connection between autophagy and sugar homeostasis. Yet, the role of autophagy in the interplay between sugar homeostasis maintenance and the sugar excess response remains ill-defined. Thus, this work addressed this issue by growing WT and *atg* mutant plants under various sucrose concentrations and examined the differential response of roots and shoots.

The presented results demonstrated that autophagy plays a role in the root response to sucrose excess. Roots of *atg* mutant plants were less affected by exogenous sucrose excess and displayed a longer primary root than WT plants (Figure 1 and Figure S1). In line with the many works discussing the importance of sugars in inhibiting seed germination [79,80,81], the current results showed that high sucrose levels indeed inhibited germination (Figure 2A and Figure S2). However, germination rate inhibition was similar between WT and *atg* mutants, suggesting that the response is root-specific. While no visible changes were noted in lateral roots, a more extensive analysis is still required.

Interestingly, a GFP-release assay revealed that autophagy is activated by excess sucrose in an organ-specific manner, with upregulation in the shoots but maintained basal levels in the roots (Figure 3A,B and Figure S4). In a recent study, Huang et al. observed reduced autophagic activity in the roots after 24 h under glucose excess, with a return to basal levels within 48 h of challenge [39]. The present analysis was conducted 10 days post-imbibition, suggesting we may have missed this transient activation. In light of these findings, sucrose excess can be used as a model to study autophagy regulation and activity in a tissue-specific manner. In addition, NBR1 was found to both mediate ABA signaling and affect lateral root initiation [50]. In contrast, the current analysis found no linkage between the response to sucrose excess and NBR1 (Figure 3C,D), suggesting the involvement of bulk autophagy. Yet, the involvement of selective autophagy governed by different receptors, as alluded by our proteomics analysis, cannot be ruled out.

Root elongation is tightly connected to carbon availability. It has long been established that sugars could affect the extension of the primary and lateral roots [82], with each sugar eliciting a unique root development response [11,12,17,18,19,37,38]. The present work showed that the reduced root sensitivity of *atg* mutants is unique to sucrose and glucose, while mannitol and fructose led to immediate root elongation arrest of all genotypes (Figure 2B–D). Coupled with the fact that no alterations were observed between the *atg* plants and WT in starch accumulation (Figure 4), it is suggested that the altered response of *atg* mutants to sucrose excess does not stem from the role of sucrose as an energy source but rather as a signaling molecule. Our metabolic profiling further confirmed this assumption, as changes in metabolites in the roots under sucrose excess were very mild (Figure 5).

The roots of hexokinase1-overexpressing plants (HXK1-OE) were highly sensitive to glucose excess, but a cross between HXK1-OE and *atg* mutant plants rescued the root phenotype [39]. This observation suggests that the response observed in *atg* mutants is HXK-dependent, meaning that it is glucose-mediated. Yet, the possibility that the responses to sucrose and glucose yield the same morphological phenotype but are distinct in their mechanism cannot be ruled out.

The extensive proteomic analysis performed here to better understand the molecular mechanism by which autophagy mediates excess sucrose response revealed differences between the lines and treatments. Interestingly, lack of autophagy had a more profound influence on the proteome, with clustering being more affected by the lines compared with the treatments (Figure 6A). Indeed, clustering of the data revealed that samples first clustered by genotype and only then by treatment (Figure S6A), further strengthening the above claim. The influence of autophagy on the proteome is a well-documented phenomenon and was also demonstrated under fixed carbon starvation and fixed nitrogen starvation [8,9]. Mining of the proteomics data by performing a biological processes analysis using David bioinformatics tools discovered many pathways altered in *atg* mutants as compared to WT plants (Figure 7, Table 2 and Table 3). Accumulation of autophagy-related proteins in *atg* mutants, and specifically NBR1, was expected due to the accumulation of autophagy proteins that could not be degraded in the mutants, as previously reported [49,83,84].

Huang et al. [39] previously demonstrated lower levels of ROS in the root tips of *atg* mutant plants exposed to glucose excess, which was proposed as the leading cause for the observed phenotype. The present work also noted an accumulation of proteins related to hydrogen peroxide processes (Figure 7A), particularly peroxidases (Figure 8A, Table 2, Supplementary Datas S2 and S3). Peroxidases have been demonstrated to lower the accumulation of ROS under aluminum stress [85]. Assessment of peroxidase activity further confirmed that under sucrose excess, peroxidases are more active in *atg* mutants, leading to lower levels of ROS (Figure 8B). Metabolic profiling of the roots also showed that trehalose accumulated in *atg* mutants under sucrose excess. Overexpression of TPS1, which is involved in trehalose synthesis, was found to partake in oxidative stress responses [56]. Trehalose might thus also be involved in the root ROS response. Our proteomics results showed a significant accumulation of TPS10 and TPS5 and a non-significant accumulation of TPS1 under 1% sucrose (Supplementary Data S3). Under 3% sucrose, TPS7 was significantly upregulated, alongside a non-significant accumulation of TPS10 (Supplementary Data S2). No accumulation of trehalose was observed under 1% sucrose in *atg* mutants (Figure 5B), suggesting that this response is also affected by other mechanisms. Overall, further examination of the connection between TPS and autophagy should be conducted.

Mitochondrial uncouplers have been found essential for maintaining ROS homeostasis [61,62,63,64]. PUMP5, a mitochondrial uncoupler protein, accumulated massively in *atg* mutant plants under sucrose excess (Table 2), suggesting its possible role in ROS homeostasis, with possible mitochondrial involvement in ROS production and response under excess sucrose. Furthermore, a recent study found that autophagy can degrade depolarized mitochondria in response to uncouplers in *Arabidopsis* roots [86]. In addition to the accumulation of PUMP5, accumulation of TCA cycle proteins was also observed in *atg* mutant plants under sucrose excess. These included succinyl CoA ligase, which was not significant but indeed showed higher levels in the mutants, citrate synthase, and succinate dehydrogenase. Succinate dehydrogenase assembly factor 2 has been demonstrated to be vital for normal root elongation in *Arabidopsis* [87], while succinyl CoA ligase was shown to be damaged by oxidative stress [88]. In addition, we previously demonstrated the upregulation of citrate synthase gene expression in *atg* mutants under dark-induced senescence [6]. Taken together, the altered profile of TCA-related proteins may underlie the root formation phenotype associated with sucrose excess.

FBA4 proved highly upregulated in *atg5-1* roots under 1% sucrose (Supplementary Data S3), and to a lower extent under 3% (Supplementary Data S2). Since FBAs are linked with sugar signaling, ABA-mediated stress [66], and the hydrogen peroxide response [89], it is tempting to assume that FBA4 participates in the sucrose-mediated response. Our results demonstrate a strong correlation between autophagy and FBA4 regulation, but the nature of this connection, both under favorable conditions and under sucrose excess conditions, remains to be further studied.

Previous data provided evidence of the upregulation of peroxisomal activity under glucose excess [39]. Examination of peroxisomal proteins in the current study indeed identified their accumulation in *atg* mutant plants under sucrose excess (Table 2). Many works characterized the role of autophagy in selective peroxisomal degradation [40,41,42], and previous research also demonstrated that autophagy is vital for the degradation of peroxisomes under abiotic stress [6,39,90,91]. Taken together, we postulate that pexophagy is involved in the plant response to sucrose excess.

Huang et al. further demonstrated upregulation of auxin, a vital root growth regulator, under glucose excess, regulated by ABCD1 [39]. ABCD1 is a peroxisomal protein essential for the conversion of indole3-butyric acid (IBA) to indole-3-acetic acid (IAA) [92]. Our analysis showed no alteration in ABCD1 levels (Supplementary Data S2), suggesting this response to be glucose-specific. Nonetheless, the proteomic analysis revealed an accumulation of IAA-amino acid conjugate hydrolases (ILLs, Table 2). Auxin conjugated to an amino acid was found to antagonize auxin activity [60,93], while ILLs can hydrolyze the conjugated amino acid, leading to an increase in bioactive auxin. Our results suggest another source for auxin accumulation in *atg* mutant plants. Besides bioavailable auxin, a decrease in PILS5 was also observed under 3% (Table 3). PILS5 is an auxin carrier that controls intracellular auxin accumulation at the ER, leading to lower cellular levels of auxin [73,74]. This might explain the higher auxin levels observed in *atg* mutants under glucose excess [39]. PILS6 was found to regulate organ growth in *Arabidopsis*, with better root growth observed in *pils6* plants [94].

**Figure 9:**
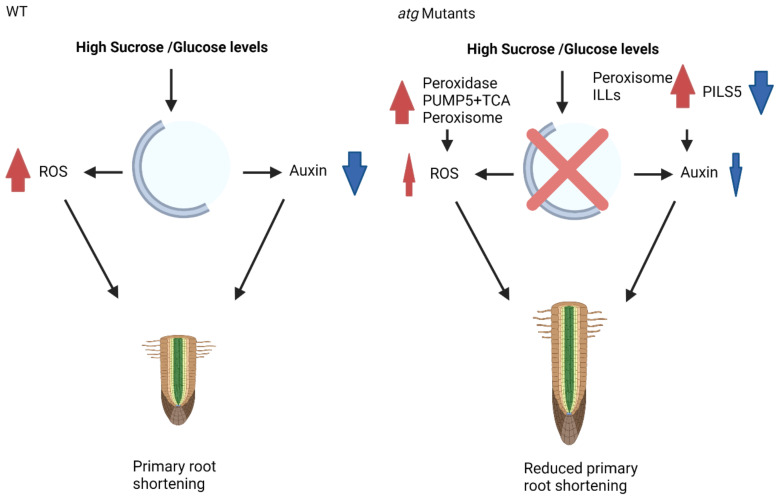
Proposed mechanism of action for autophagy during sucrose excess. In WT plants under high sucrose/glucose levels, primary root shortening occurs via the accumulation of ROS and reduced levels of auxin. In contrast, under similar conditions, *atg* mutant plants present reduced ROS accumulation, which is mediated via peroxidases, PUMP5, TCA cycle enzymes, and peroxisomal activity. There is also less auxin reduction, which is mediated by peroxisomal proteins and ILL activity. These changes in protein and organellar activity lead to reduced primary root shortening. Red arrows indicate upregulation, blew arrows indicate downregulation.

To conclude, previous analyses suggested peroxisomal activity as a modulator of ROS and as a primary source of bioavailable auxin under glucose excess, leading to the lower sensitivity of *atg* mutant plants to glucose-mediated root growth inhibition [39]. The current analysis demonstrated that a similar response occurs under sucrose excess and is regulated by basal levels of autophagy or mediated by enhanced autophagic activity in the shoots via root-shoot communication. This response is more complex than previously described and relies on many other cellular components, including mitochondrial and cellular proteins. Such a response reduces ROS accumulation and auxin decline, leading to a less pronounced root phenotype in *atg* mutants (Figure 9).

## 4. Materials and Methods

### 4.1. Plant Lines

*Arabidopsis thaliana* (*Arabidopsis*) ecotype Columbia (Col-0) was used in this study. The lines used were as follows: *atg5-1* (SAIL_129B079) [95], *atg7-2* (GK-655B06) [95], and GFP-ATG8f-HA [96]. NBR1 OX (NBR1-TAP) and *nbr1* KO lines [50] were generously provided by Professor A. Sirko, Institute of Biochemistry and Biophysics, Polish Academy of Sciences.

### 4.2. Plant Growth Conditions

Seeds were surface-sterilized using vapor-phase sterilization with Cl_2_ gas for 3 h, and sown on 1% (*w*/*v*) agar plates containing Nitsch medium (Duchefa Biochemia, Haarlem, Netherlands) and varying concentrations (*w*/*v*) of sucrose: 0%, 1% (29 mM), 2% (58 mM), 3% (87 mM), and 4% (116 mM). Plates containing glucose, fructose, or mannitol contained the molar equivalent to the sucrose concentration. Plates were then imbibed for 72 h at 4 ℃, in the dark, and grown vertically (except for the starch assay, which was grown horizontally) in a growth chamber (FITOCLIMA S600PLH Aralab climatic chamber, Rio de Mouro, Portugal), under 22 ℃, 50% Rh, and constant light (125 µmol/m^2^/s), for 10 or 14 days.

### 4.3. Root Elongation Analysis

For root growth measurements, 14-day-old seedlings grown on Nitsch plates were photographed, and the root length was measured using ImageJ analysis software [97]. To measure the root growth rate, the tip of the root was marked every 48 h for 10 days. The plates were photographed, and the root length was measured using ImageJ. Each plate contained all the measured genotypes to ensure similar growth conditions. At least three plates were prepared for each analysis, with a varied number of plants per line in each plate. Dunnett’s test was conducted to compare the tested lines with the WT under each treatment. Data for root length measurements are displayed as box and whiskers plots, with error bars indicating minimum and maximum values. Data for the root growth rate are displayed as line graphs, with error bars indicating the standard error (SE).

### 4.4. Seed Germination Analysis

*Arabidopsis* seeds were sown on Nitsch plates. After 48 h of imbibition, the plates were transferred to a growth chamber, and seed germination, scored by radicle protrusion, was monitored at several time points (15, 19, 24, 36, and 42 h post-imbibition). Four to six plates were examined, and a total of 134–289 seeds were counted.

### 4.5. Protein Extraction for Immunoblot

Leaf and root tissues (~100 mg) from 10-day-old plants were ground in a bead-beater and proteins were extracted using extraction buffer (50 mM Tris-HCl pH 7.5, 20 mM NaCl, 10% glycerol, 1% Triton, and a 1:7 ratio of protease inhibitor; 04693124001, Roche, Basel, Switzerland), based on a previously described extraction buffer [98]. Samples were then centrifuged for 15 min at 20,000 g, at 4 °C, and the supernatant was collected and stored at −20 °C.

### 4.6. Immunoblot Analysis

Total proteins were separated using a 12.5% SDS-PAGE, under a Tris-glycine running buffer [99]. The proteins were then transferred to a 0.2 µm polyvinylidene difluoride membrane (Amersham Hybond, GE Healthcare Life Science, Amersham, UK) under a transfer buffer [100]. Coomassie brilliant blue (0.25% *w*/*v* in destain solution: 50% methanol, 10% acetic acid, 40% DDW, Raymond A. Lamb) was used to ensure equal loading. For immunodetection, membranes were incubated with rabbit polyclonal anti-GFP antibodies (AB290, Abcam, Cambridge, UK) and later with goat anti-rabbit antibody (A00098, GenScript, Piscataway, NJ, USA). Proteins were then imaged using the Gel Imager (Fusing FX, Vilber Lumart, Collegien, France).

### 4.7. Starch Staining

Seedlings grown for 10 days on Nitsch medium with various sucrose concentrations were collected into 2-mL tubes (the entire plant was collected). Starch was stained by incubating the plants for 10 min in Lugol’s IKI solution, as previously described [101]. Pictures were taken using a stereoscope binocular (KERN Optics, Microscope VIS software(basic), Balingen, Germany).

### 4.8. Starch Quantification

Seedlings were grown on Nitsch medium with 0%, 1%, or 3% sucrose as described above. Fresh plant tissue (20 mg) of 10-day-old seedlings was then collected. Starch extraction was performed according to previously published protocols [102]. The starch content was then quantified using a Starch Assay Kit (Abcam©, ab83393, Cambridge, UK). Data are displayed as box and whiskers plots, with error bars indicating minimum and maximum values.

### 4.9. GC-MS Analysis

Metabolites were extracted following an established gas chromatography-mass spectrometry (GC-MS)-based metabolite profiling protocol [103], with slight modifications. Plant samples (10-days-old roots and shoots separately, 10–30 mg per sample), grown vertically on Nitsch plates containing various sucrose concentrations, were collected in 2-mL tubes (4–6 biological replicated per line per treatment). The samples were flash-frozen in liquid nitrogen and stored at −80 °C until extraction. The samples were extracted in methanol containing an internal standard (0.2 mg ribitol mL^−1^ water), shaken for 15 min at 70 °C, and centrifuged at 14,000 rpm for 10 min. The supernatant was transferred to new tubes, and chloroform and double-distilled water were added. Tubes were then centrifuged at 14,000 rpm for 15 min, and aliquots of 150 μL of the upper phase of each sample were transferred to new 1.5 mL tubes and dried by Speedvac (Concentrator Plus, Eppendorf, Hamburg, Germany), overnight. Derivatization was carried out as previously described [103].

Polar metabolites were measured by the Agilent 7200B GC/Q-TOF. The injection and separation procedures were performed according to Dahtt using the DB-35MS column [104]. Metabolite detection and annotation were performed by Quant software (Agilent, Santa Clara, CA, USA), according to an accurate mass library of known markers generated by our group and run in the same system. More information can be found in Supplementary Table S1. Following blank subtraction, the peak area of each metabolite was normalized to the internal standard (i.e., ribitol) in each sample, and by the fresh weight of the sample.

### 4.10. Protein Extraction for Shotgun Proteomics

Proteins were extracted from 10-day-old seedlings (*atg5-1* and WT) grown on 1% and 3% sucrose Nitsch plates. Protein extraction was performed according to Rodiger et al. [105]. Four biological replicates were tested for each treatment and line.

### 4.11. Sample Preparation for LC-MS/MS Analysis

Samples in extraction buffer (40 mM Tris-HCl pH 6.8, 10% glycerol with protease inhibitor mix) were supplemented with 4% sodium dodecyl sulfate and 20 mM dithiothreitol and heated at 70 °C for 10 min. Protein (25 µg) was precipitated using the chloroform/methanol method [106] and then solubilized in 100 μL of a solution containing 8 M urea, 10 mM DTT, and 25 mM Tris-HCl pH 8.0, and incubated for 30 min at 22 °C. Iodoacetamide (55 mM) was added, after which samples were incubated for 30 min (22 °C, in the dark). Then, DTT (10 mM) was added. After dilution with 7 volumes of 25 mM Tris-HCl pH 8.0, sequencing-grade modified trypsin (Promega Corp., Madison, WI, USA) was added (0.4 μg/sample), followed by incubation overnight at 37 °C with agitation. The samples were acidified by the addition of 0.2% formic acid and desalted on C18 homemade Stage tips. The peptide concentration was determined by the absorbance (280 nm) of 0.75 µg peptides injected into the mass spectrometer.

### 4.12. NanoLC-MS/MS Analysis

Mass spectrometry analysis was performed using a Q Exactive-HF mass spectrometer (Thermo Fisher Scientific, Waltham, MA, USA) coupled online to a nanoflow UHPLC instrument, Ultimate 3000 Dionex (Thermo Fisher Scientific, Waltham, MA, USA). Peptides dissolved in 0.1% formic acid were separated without a trap column over a 120-min acetonitrile gradient run at a flow rate of 0.3 μL/min on a reverse-phase 25-cm-long C18 column (75 μm ID, 2 μm, 100 Å, Thermo PepMapRSLC). The instrument settings were as described by Scheltema et al. [107] Survey scans (300–1650 *m*/*z*, target value 3 × 10^6^ charges, maximum ion injection time 20 ms) were acquired and followed by higher-energy collisional dissociation (HCD)-based fragmentation (normalized collision energy 27). A resolution of 60,000 was used for survey scans, and up to 15 dynamically chosen, most abundant precursor ions, with a “peptide-preferable” profile, were fragmented (isolation window 1.8 *m*/*z*). The MS/MS scans were acquired at a resolution of 15,000 (target value 1 × 10^5^ charges, maximum ion injection times 25 ms). Dynamic exclusion was 20 s. Data were obtained using Xcalibur software (Thermo Scientific, Waltham, MA, USA). To avoid carryover, the column was washed between samples with 80% acetonitrile and 0.1% formic acid, for 25 min.

### 4.13. NanoLC-MS/M.S. Data Analysis

Mass spectra data were processed using the MaxQuant computational platform, version 1.6.17.0. Peak lists were searched against the Uniprot *Arabidopsis* FASTA sequence database from 21 March 2021, containing 17,898 entries. The search included cysteine carbamidomethylation as a fixed modification, and N-terminal acetylation and oxidation of methionine as variable modifications, with up to two miscleavages allowed. The match-between-runs option was used. Peptides with a length of at least seven amino acids were considered, and the required FDR was set to 1% at the peptide and protein levels. Relative protein quantification in MaxQuant was performed using the label-free quantification (LFQ) algorithm [108]. Statistical analysis (*n* = 4) was performed using the Perseus statistical package [109]. Only proteins for which at least three valid LFQ values were obtained in at least one sample group, were accepted for statistical analysis by volcano plot (Student’s *t*-test, *p* < 0.05). After application of this filter, a random value was substituted for proteins for which LFQ could not be determined (“Imputation” function of Perseus). The imputed values were in the range of 10% of the median value of all the proteins in the sample and allowed for the calculation of *p* values.

### 4.14. Proteomics and Metabolomics Data Visualization

Proteomics PCA, heatmap, and volcano plots were generated using RStudio [110]. Readxl [111], ggbiplot [112], ggplot2 [113], and ggrepel [114] packages were used for the plots. PCA was conducted on the raw LFQ values, which were then centered using the R function ‘scale’. Heat maps were prepared using the average LFQ of each protein; the LFQ values were first centered using the R function ‘scale’. All proteins with a zero value were excluded from the analysis.

Volcano plots were generated from the log2 fold change extracted from the MaxQuant analysis. The threshold for upregulated or downregulated proteins was a fold-change above log_10_(0.6) or below log_10_(−0.6) and a *p*-value below 0.05. The code was taken and modified from https://biocorecrg.github.io/CRG_RIntroduction/volcano-plots.html (accessed on 26 December 2021). Venn diagrams were generated using the http://bioinformatics.psb.ugent.be/webtools/Venn/ (accessed on 26 December 2021). The protein list for the diagram was the one achieved from the volcano plot R code. Functional annotation clustering of the upregulated and downregulated proteins was performed using the David Bioinformatics Resource [115]. The protein list for the diagram was the one achieved from the volcano plot R code. The GOTERM_BP_DIRECT category was used, and processes with a *p*-value of 0.05 or below were chosen.

Metabolomics PCA was performed using RStudio on the raw area values, which were centered using the R function ‘scale’. Heat maps were prepared from the log2 values normalized to WT 0%, and visualized using the Multi-Experiment Viewer (MeV) [116].

### 4.15. Peroxidase Activity Assay

Plant root samples (10–30 mg tissue) of 10-day-old plants grown under various sucrose concentrations were collected, snap-frozen in liquid nitrogen, and stored at −80 °C. Samples were extracted using Amplex ×1 reaction buffer (Amplex™ Red Hydrogen Peroxide/Peroxidase Assay Kit, Invitrogen, Waltham, MA, USA), and peroxidase activity was assessed according to the kit protocol. Samples were diluted with the Amplex reaction buffer (1 µL of sample in 49.5 µL of reaction buffer) into a 96-well plate, and then further diluted with 50 µL working solution (50 µL 10 mM Amplex red reagent, 500 µL 20 mM hydrogen peroxide, 4.45 mL 1× reaction buffer). After adding the working solution, the plate was incubated at room temperature for 30 min in the dark, and absorbance was measured at 560 nm. Samples containing 0.5, 1, 2, and 4 milli-units of horseradish peroxidase (HRP) were used for a calibration curve, as well as a blank measurement of reaction buffer and working solution.

## Figures and Tables

**Table 1:** Metabolic profiling of 10-day-old seedling roots under 3% sucrose.

Group	Metabolite	WT	*atg5-1*	*atg7-2*
Amino acids	Aspartic acid	2.67 ± 0.25	2.68 ± 0.52	3.58 ± 0.55
	Arginine	0.001 ± 0.0003	0.0009 ± 0.0002	**0.003 ± 0.0007**
	Glutamic acid	1.70 ± 0.25	4.16 ± 1.25	3.27 ± 0.47
	Glycine	3.95 ± 0.19	3.27 ± 0.32	5.06 ± 0.77
	Methionine	1.60 ± 0.19	**3.00 ± 0.50**	**3.11 ± 0.21**
	Ornithine	27.47 ± 3.78	31.02 ± 8.29	39.41 ± 3.85
	Phenylalanine	0.61 ± 0.15	0.84 ± 0.19	0.77 ± 0.08
	Proline	1.3 ± 0.77	0 ± 0	**3.68 ± 0.82**
	Serine	4.91 ± 0.73	3.43 ± 0.46	3.40 ± 0.46
	Threonine	20.24 ± 1.80	**14.51 ± 1.86**	15.95 ± 1.09
	Tyrosine	3.07 ± 0.21	3.21 ± 0.50	**5.40 ± 0.60**
Organic acids	Citric acid	216.59 ± 35.00	274 ± 63.07	344.79 ± 47.37
	Fumaric acid	17.16 ± 0.84	16.63 ± 2.27	22.78 ± 2.45
	Alpha ketoglutaric acid	7.17 ± 0.70	54.71 ± 22.09	53.68 ± 15.69
	Malic acid	198.42 ± 12.77	174.23 ± 18.56	163.00 ± 17.24
	Succinic acid	107.84 ± 7.01	**58.04 ± 9.11**	84.73 ± 11.61
Sugars	Alpha arabinose	5.15 ± 0.67	5.55 ± 0.82	6.70 ± 0.72
	Fructose	308.42 ± 44.90	345.81 ± 69.52	437.51 ± 45.51
	Glucose	2.16 ± 0.31	2.54 ± 0.51	3.25 ± 0.38
	Lyxose/Xylose	4.15 ± 1.32	4.50 ± 1.10	6.57 ± 0.72
	Myo inositol	35.10 ± 5.59	33.40 ± 6.87	43.05 ± 4.49
	Sucrose	387.40 ± 84.35	340.92 ± 69.81	373.64 ± 49.81
	Trehalose	5.64 ± 5.64	**453.75 ± 106.12**	**252.63 ± 33.04**
Others	Phosphoric acid	6.06 ± 0.92	4.51 ± 0.74	5.55 ± 0.58

Values represent the average ± standard error (*n* = 5–6). Values were all normalized to the average WT 0% and presented at relative levels. Significant differences (*p* < 0.05) as compared with WT under the same treatment, calculated by Dunnett’s test, are highlighted in bold.

**Table 2:** Selected proteins upregulated in roots of *atg5-1* seedlings under different sucrose treatments.

Biological Process	Protein Name	Fold Change (Compared to WT)	*p* Value (Compared to WT)
		1%/3%	1%/3%
Hydrogen peroxide catabolic processes	Peroxidase 15	2.4/1.7	0.0179/0.0365
	Peroxidase 23	1.4/1.6	0.2036/0.0270
	Peroxidase 51	NA/1.9	NA/0.0289
	Cationic amino acid transporter 2	2.3/1.3	0.0212/0.0271
Tricarboxylic acid cycle	Succinyl-coA ligase, alpha subunit	2.2/3.3	0.2514/0.1600
	Citrate synthase 3	1.3/1.5	0.0067/0.0041
	Succinate dehydrogenase subunit 7B	1.9/8.0	0.5115/0.0146
Mitochondrial proteins	Mitochondrial uncoupling protein 5	3.4/20.9	0.4634/0.0255
Peroxisomal proteins	Citrate synthase 2	1.1/1.3	0.2547/0.0076
	Acyl-coenzyme A oxidase 1.2	1.2/1.5	0.066/0.0038
	Coenzyme A oxidase 2	1.1/1.2	0.1495/0.0118
	Coenzyme A oxidase 1	1.2/1.3	0.0318/0.0075
	Coenzyme A oxidase 3	1.1/1.2	0.3331/0.0116
	Coenzyme A oxidase 4	1.2/1.2	0.0476/0.0117
	Fatty acid beta-oxidation-multifunctional protein 2	1.2/1.2	0.0286/0.0056
	Malate dehydrogenase	1.2/1.2	0.0417/0.0101
	Long chain acyl-coA synthetase 8	1.3/1.4	0.0009/0.0026
Auxin processing	IAA-amino acid hydrolase ILR1-like 4	1.3/1.5	0.0048/0.0017
	IAA-amino acid hydrolase ILR1-like 1	1.6/1.4	0.0056/0.0034
	IAA-amino acid hydrolase ILR1-like 2	1.3/1.6	0.0304/0.0054
	Indole-3-acetic acid-amido synthetase GH3.17	1.4/1.7	0.0006/0.0009
Sucrose-related processes	Sucrose synthase 1	1.3/1.7	0.0005/0.0370
	Sucrose synthase 4	1.5/2.2	0.0045/0.0101
	Sucrose synthase 3	1.5/1.5	0.0248/0.0374

**Table 3:** Selected proteins downregulated in roots of *atg5-1* seedlings under different sucrose treatments.

Biological Process	Protein Name	Fold Change (Compared to WT) 1%/3%	*p* Value (Compared to WT) 1%/3%
Auxin transport	PIN LIKE 5 (PILS5)	1.3/0.3	0.4539/0.0055
Cell-wall responses	Expansin-A11	0.2/0.6	0.1001/0.0006
	Expansin-A1	0.7/0.6	0.3445/0.0081

## Data Availability

Not applicable.

## Supplementary Materials

The following supporting information can be downloaded at: https://www.mdpi.com/article/10.3390/ijms23073891/s1.

## References

[1] Bassham D.C., Laporte M., Marty F., Moriyasu Y., Ohsumi Y., Olsen L.J., Yoshimoto K. (2006). Autophagy in development and stress responses of plants. Autophagy.

[2] Michaeli S., Galili G., Genschik P., Fernie A.R., Avin-Wittenberg T. (2016). Autophagy in Plants—What’s New on the Menu?. Trends Plant Sci..

[3] Avin-Wittenberg T., Baluška F., Bozhkov P.V., Elander P.H., Fernie A.R., Galili G., Hassan A., Hofius D., Isono E., Le Bars R. (2018). Autophagy-related approaches for improving nutrient use efficiency and crop yield protection. J. Exp. Bot..

[4] Izumi M., Hidema J., Makino A., Ishida H. (2013). Autophagy contributes to nighttime energy availability for growth in Arabidopsis. Plant Physiol..

[5] Barros J.A.S., Cavalcanti J.H.F., Medeiros D.B., Nunes-Nesi A., Avin-Wittenberg T., Fernie A.R., Araujo W.L. (2017). Autophagy deficiency compromises alternative pathways of respiration following energy deprivation in *Arabidopsis thaliana*. Plant Physiol..

[6] Barros J.A.S., Magen S., Lapidot-Cohen T., Rosental L., Brotman Y., Araújo W.L., Avin-Wittenberg T. (2021). Autophagy is required for lipid homeostasis during dark-induced senescence. Plant Physiol..

[7] Fan J., Yu L., Xu C. (2019). Dual role for autophagy in lipid metabolismin arabidopsis. Plant Cell.

[8] McLoughlin F., Augustine R.C., Marshall R.S., Li F., Kirkpatrick L.D., Otegui M.S., Vierstra R.D. (2018). Maize multi-omics reveal roles for autophagic recycling in proteome remodelling and lipid turnover. Nat. Plants.

[9] McLoughlin F., Marshall R.S., Ding X., Chatt E.C., Kirkpatrick L.D., Augustine R.C., Li F., Otegusi M.S., Vierstra R.D. (2020). Autophagy plays prominent roles in amino acid, nucleotide, and carbohydrate metabolism during fixed-carbon starvation in maize. Plant Cell.

[10] Avin-Wittenberg T., Bajdzienko K., Wittenberg G., Alseekh S., Tohge T., Bock R., Giavalisco P., Fernie A.R. (2015). Global analysis of the role of autophagy in cellular metabolism and energy homeostasis in arabidopsis seedlings under carbon starvation. Plant Cell.

[11] Wind J., Smeekens S., Hanson J. (2010). Sucrose: Metabolite and signaling molecule. Phytochemistry.

[12] John R., Raja V., Ahmad M., Jan N., Majeed U., Ahmad S., Yaqoob U., Kaul T. (2016). Trehalose: Metabolism and role in stress signaling in plants. Stress Signal. Plants Genom. Proteom. Perspect..

[13] Meng L.S., Xu M.K., Wan W., Yu F., Li C., Wang J.Y., Wei Z.Q., Lv M.J., Cao X.Y., Li Y.Z. (2018). Sucrose signaling regulates anthocyanin biosynthesis through a MAPK cascade in *Arabidopsis thaliana*. Genetics.

[14] Teng S., Keurentjes J., Bentsink L., Koornneef M., Smeekens S. (2005). Sucrose-specific induction of anthocyanin biosynthesis in Arabidopsis requires the MYB75/PAP1 gene. Plant Physiol..

[15] Malamy J.E., Ryan K.S. (2001). Environmental regulation of lateral root initiation in Arabidopsis. Plant Physiol..

[16] Gupta A., Singh M., Laxmi A. (2015). Interaction between glucose and brassinosteroid during the regulation of lateral root development in arabidopsis. Plant Physiol..

[17] Zhong Y., Xie J., Wen S., Wu W., Tan L., Lei M., Shi H., Zhu J.K. (2020). TPST is involved in fructose regulation of primary root growth in *Arabidopsis thaliana*. Plant Mol. Biol..

[18] Sakr S., Wang M., Dédaldéchamp F., Perez-Garcia M.D., Ogé L., Hamama L., Atanassova R. (2018). The sugar-signaling hub: Overview of regulators and interaction with the hormonal and metabolic network. Int. J. Mol. Sci..

[19] Rolland F., Baena-Gonzalez E., Sheen J. (2006). Sugar sensing and signaling in plants: Conserved and novel mechanisms. Annu. Rev. Plant Biol..

[20] Overvoorde P., Fukaki H., Beeckman T. (2010). Auxin control of root development. Cold Spring Harb. Perspect. Biol..

[21] Ljung K., Nemhauser J.L., Perata P. (2015). New mechanistic links between sugar and hormone signalling networks. Curr. Opin. Plant Biol..

[22] Laby R.J., Kincaid M.S., Kim D., Gibson S.I. (2000). The *Arabidopsis* sugar-insensitive mutants *sis4* and *sis5* are defective in abscisic acid synthesis and response. Plant J. Cell Mol. Biol..

[23] Arenas-Huertero F., Arroyo A., Zhou L., Sheen J., Leó P. (2000). Analysis of *Arabidopsis* glucose insensitive mutants, *gin5* and *gin6*, reveals a central role of the plant hormone ABA in the regulation of plant vegetative development by sugar. Genes Dev..

[24] Kushwah S., Jones A.M., Laxmi A. (2011). Cytokinin interplay with ethylene, auxin, and glucose signaling controls arabidopsis seedling root directional growth. Plant Physiol..

[25] Kushwah S., Laxmi A. (2014). The interaction between glucose and cytokinin signal transduction pathway in *Arabidopsis thaliana*. Plant Cell Environ..

[26] Yoon J., Cho L.H., Tun W., Jeon J.S., An G. (2021). Sucrose signaling in higher plants. Plant Sci..

[27] Lunn J.E., Feil R., Hendriks J.H.M., Gibon Y., Morcuende R., Osuna D., Scheible W.R., Carillo P., Hajirezaei M.R., Stitt M. (2006). Sugar-induced increases in trehalose 6-phosphate are correlated with redox activation of ADPglucose pyrophosphorylase and higher rates of starch synthesis in *Arabidopsis thaliana*. Biochem. J..

[28] Nunes C., O’Hara L.E., Primavesi L.F., Delatte T.L., Schluepmann H., Somsen G.W., Silva A.B., Fevereiro P.S., Wingler A., Paul M.J. (2013). The trehalose 6-phosphate/snRK1. signaling pathway primes growth recovery following relief of sink limitation. Plant Physiol..

[29] Yadav U.P., Ivakov A., Feil R., Duan G.Y., Walther D., Giavalisco P., Piques M., Carillo P., Hubberten H.M., Stitt M. (2014). The sucrose-trehalose 6-phosphate (Tre6P) nexus: Specificity and mechanisms of sucrose signalling by Tre6P. J. Exp. Bot..

[30] Gómez L.D., Baud S., Gilday A., Li Y., Graham I.A. (2006). Delayed embryo development in the *ARABIDOPSIS TREHALOSE-6-PHOSPHATE SYNTHASE 1* mutant is associated with altered cell wall structure, decreased cell division and starch accumulation. Plant J..

[31] Zhang Y., Primavesi L.F., Jhurreea D., Andralojc P.J., Mitchell R.A.C., Powers S.J., Schuluepmann H., Delatte T., Wingler A., Paul M.J. (2009). Inhibition of SNF1-related protein kinasel activity and regulation of metabolic pathways by trehalose-6-phosphate1. Plant Physiol..

[32] Martínez-Barajas E., Delatte T., Schluepmann H., de Jong G.J., Somsen G.W., Nunes C., Primavesi L.F., Coello P., Mitchell A.C.R., Paul M.J. (2011). Wheat grain development is characterized by remarkable trehalose 6-phosphate accumulation pregrain filling: Tissue distribution and relationship to SNF1-related protein kinase1 activity. Plant Physiol..

[33] Baena-González E., Lunn J.E. (2020). SnRK1 and trehalose 6-phosphate—Two ancient pathways converge to regulate plant metabolism and growth. Curr. Opin. Plant Biol..

[34] Wurzinger B., Nukarinen E., Nägele T., Weckwerth W., Teige M. (2018). The SnRK1 Kinase as Central Mediator of Energy Signaling between Different Organelles. Plant Physiol..

[35] Im J.H., Cho Y.H., Kim G.D., Kang G.H., Hong J.W., Yoo S.D. (2014). Inverse modulation of the energy sensor Snf1-related protein kinase 1 on hypoxia adaptation and salt stress tolerance in *Arabidopsis thaliana*. Plant Cell Environ..

[36] Baena-González E., Rolland F., Thevelein J.M., Sheen J. (2007). A central integrator of transcription networks in plant stress and energy signalling. Nature.

[37] Cho Y.H., Yoo S.D. (2011). Signaling role of fructose mediated by FINS1/FBP in *Arabidopsis thaliana*. PLoS Genet..

[38] Valifard M., le Hir R., Müller J., Scheuring D., Neuhaus H.E., Pommerrenig B. (2021). Vacuolar fructose transporter SWEET17 is critical for root development and drought tolerance. Plant Physiol..

[39] Huang L., Yu L.J., Zhang X., Fan B., Wang F.Z., Dai Y.S., Qi H., Zhou Y., Xie L.J., Xiao S. (2019). Autophagy regulates glucose-mediated root meristem activity by modulating ROS production in *Arabidopsis*. Autophagy.

[40] Farmer L.M., Rinaldi M.A., Young P.G., Danan C.H., Burkhart S.E., Bartel B. (2013). Disrupting autophagy restores peroxisome function to an *Arabidopsis lon2* mutant and reveals a role for the LON2 protease in peroxisomal matrix protein degradation. Plant Cell.

[41] Kim J., Lee H., Lee H.N., Kim S.H., Shin K.D., Chung T. (2013). Autophagy-related proteins are required for degradation of peroxisomes in *Arabidopsis* hypocotyls during seedling growth. Plant Cell.

[42] Shibata M., Oikawa K., Yoshimoto K., Kondo M., Mano S., Yamada K., Hayashi M., Sakamoto W., Ohsumi Y., Nishimura M. (2013). Highly oxidized peroxisomes are selectively degraded via autophagy in *Arabidopsis*. Plant Cell.

[43] Chung T., Phillips A.R., Vierstra R.D. (2010). ATG8 lipidation and ATG8-mediated autophagy in Arabidopsis require ATG12 expressed from the differentially controlled *ATG12A* and *ATG12B* loci. Plant J..

[44] Marshall R.S., Vierstra R.D. (2018). Autophagy: The Master of Bulk and Selective Recycling. Annu. Rev. Plant Biol..

[45] Masclaux-Daubresse C., Clément G., Anne P., Routaboul J.-M., Guiboileau A., Soulay F., Shirasu K., Yoshimoto K. (2014). Stitching together the Multiple Dimensions of Autophagy Using Metabolomics and Transcriptomics Reveals Impacts on Metabolism, Development, and Plant Responses to the Environment in Arabidopsis. Plant Cell.

[46] Minina E.A., Moschou P.N., Vetukuri R.R., Sanchez-Vera V., Cardoso C., Liu Q., Elander P.H., Dalman K., Beganovic M., Yilmaz J.L. (2018). Transcriptional stimulation of rate-limiting components of the autophagic pathway improves plant fitness. J. Exp. Bot..

[47] Klionsky D.J., Kamal Abdel-Aziz A., Abdelfatah S., Abdellatif M., Abdoli A., Abel S., Abeliovich H., Abildgaard M.H., Abudu Y.P., Acevedo-Arozena A. (2021). Guidelines for the use and interpretation of assays for monitoring autophagy. Autophagy.

[48] Avin-Wittenberg T., Michaeli S., Honig A., Galili G. (2012). ATI1, a newly identified atg8-interacting protein, binds two different Atg8 homologs. Plant Signal. Behav..

[49] Zhou J., Wang J., Cheng Y., Chi Y.J., Fan B., Yu J.Q., Chen Z. (2013). NBR1-Mediated Selective Autophagy Targets Insoluble Ubiquitinated Protein Aggregates in Plant Stress Responses. PLoS Genet..

[50] Tarnowski L., Rodriguez M.C., Brzywczy J., Piecho-Kabacik M., Krčkova Z., Martinec J., Wawrzynska A., Sirko A. (2020). A selective autophagy cargo receptor NBR1 modulates abscisic acid signalling in *Arabidopsis thaliana*. Sci. Rep..

[51] Li F., Chung T., Pennington J.G., Federico M.L., Kaeppler H.F., Kaeppler S.M., Otegui M.S., Vierstra R.D. (2015). Autophagic recycling plays a central role in maize nitrogen remobilization. Plant Cell.

[52] Yoshimoto K., Shibata M., Kondo M., Oikawa K., Sato M., Toyooka K., Shirasu K., Nishimura M., Ohsumi Y. (2014). Organ-specific quality control of plant peroxisomes is mediated by autophagy. J. Cell Sci..

[53] Kurusu T., Koyano T., Hanamata S., Kubo T., Noguchi Y., Yagi C., Nagata N., Yamamoto T., Ohnishi T., Okazaki Y. (2014). OsATG7 is required for autophagy-dependent lipid metabolism in rice postmeiotic anther development. Autophagy.

[54] Izumi M., Hidema J., Ishida H. (2013). Deficiency of autophagy leads to significant changes of metabolic profiles in *Arabidopsis*. Plant Signal. Behav..

[55] Stein O., Granot D. (2019). An overview of sucrose synthases in plants. Front. Plant Sci..

[56] Cortina C., Culiáñez-Macià F.A. (2005). Tomato abiotic stress enhanced tolerance by trehalose biosynthesis. Plant Sci..

[57] Wawrzyńska A., Sirko A. (2020). The role of selective protein degradation in the regulation of iron and sulfur homeostasis in plants. Int. J. Mol. Sci..

[58] Hirota T., Izumi M., Wada S., Makino A., Ishida H. (2018). Vacuolar protein degradation via autophagy provides substrates to amino acid catabolic pathways as an adaptive response to sugar starvation in *Arabidopsis thaliana*. Plant Cell Physiol..

[59] Bassham D.C. (2007). Plant autophagy—More than a starvation response. Curr. Opin. Plant Biol..

[60] Ruiz Rosquete M., Barbez E., Kleine-Vehn J. (2012). Cellular auxin homeostasis: Gatekeeping is housekeeping. Mol. Plant.

[61] De Arcuri L.C.M., Nunes-Laitz A.V., Lima R.P.M., Barreto P., Marinho A.N., Arruda P., Maia I.G. (2021). Knockdown of Mitochondrial Uncoupling Proteins 1 and 2 (AtUCP1 and 2) in *Arabidopsis thaliana* Impacts Vegetative Development and Fertility. Plant Cell Physiol..

[62] Nogueira F.T.S., Sassaki F.T., Maia I.G. (2011). *Arabidopsis thaliana* uncoupling proteins (AtUCPs): Insights into gene expression during development and stress response and epigenetic regulation. J. Bioenerg. Biomembr..

[63] Sweetlove L.J., Lytovchenko A., Morgan M., Nunes-Nesi A., Taylor N.L., Baxter C.J., Eickmeier I., Fernie A.R. (2006). Mitochondrial uncoupling protein is required for efficient photosynthesis. Proc. Natl. Acad. Sci. USA.

[64] Popov V.N., Syromyatnikov M.Y., Fernie A.R., Chakraborty S., Gupta K.J., Igamberdiev A.U. (2021). The uncoupling of respiration in plant mitochondria: Keeping reactive oxygen and nitrogen species under control. J. Exp. Bot..

[65] Lv G.Y., Guo X.G., Xie L.P., Xie C.G., Zhang X.H., Yang Y., Xiao L., Tang Y.Y., Pan X.L., Gou A.G. (2017). Molecular Characterization, Gene Evolution, and Expression Analysis of the Fructose-1, 6-bisphosphate Aldolase (FBA) Gene Family in Wheat (*Triticum aestivum* L.). Front. Plant Sci..

[66] Lu W., Tang X., Huo Y., Xu R., Qi S., Huang J., Zheng C., Wu C.A. (2012). Identification and characterization of fructose 1,6-bisphosphate aldolase genes in *Arabidopsis* reveal a gene family with diverse responses to abiotic stresses. Gene.

[67] Bieniawska Z., Paul Barratt D.H., Garlick A.P., Thole V., Kruger N.J., Martin C., Zrenner R., Smith A.M. (2007). Analysis of the sucrose synthase gene family in *Arabidopsis*. Plant J..

[68] Xu S.M., Brill E., Llewellyn D.J., Furbank R.T., Ruan Y.L. (2012). Overexpression of a potato sucrose Synthase gene in cotton accelerates leaf expansion, reduces seed abortion, and enhances fiber production. Mol. Plant.

[69] Yang J., Zhang G., An J., Li Q., Chen Y., Zhao X., Wu J., Wang Y., Hao Q., Wang W. (2020). Expansin gene TaEXPA2 positively regulates drought tolerance in transgenic wheat (*Triticum aestivum* L.). Plant Sci..

[70] Che J., Yamaji N., Shen R.F., Ma J.F. (2016). An Al-inducible expansin gene, *OsEXPA10* is involved in root cell elongation of rice. Plant J..

[71] Li A.X., Han Y.Y., Wang X., Chen Y.H., Zhao M.R., Zhou S.M., Wang W. (2015). Root-specific expression of wheat expansin gene *TaEXPB23* enhances root growth and water stress tolerance in tobacco. Environ. Exp. Bot..

[72] Kazibwe Z., Liu A.Y., MacIntosh G.C., Bassham D.C. (2019). The Ins and Outs of Autophagic Ribosome Turnover. Cells.

[73] Barbez E., Kubeš M., Rolčík J., Béziat C., Pěnčík A., Wang B., Rosquete M.R., Zhu J., Dobrev P.I., Lee Y. (2012). A novel putative auxin carrier family regulates intracellular auxin homeostasis in plants. Nature.

[74] Sun L., Feraru E., Feraru M.I., Waidmann S., Wang W., Passaia G., Wang Z.Y., Wabnik K., Kleine-Vehn J. (2020). PIN-LIKES Coordinate Brassinosteroid Signaling with Nuclear Auxin Input in *Arabidopsis thaliana*. Curr. Biol..

[75] Consortium T.U. (2021). UniProt: The universal protein knowledgebase in 2021. Nucleic Acids Res..

[76] Thompson A.R., Doelling J.H., Suttangkakul A., Vierstra R.D. (2005). Autophagic nutrient recycling in Arabidopsis directed by the ATG8 and ATG12 conjugation pathways. Plant Physiol..

[77] Janse van Rensburg H.C., van den Ende W., Signorelli S. (2019). Autophagy in Plants: Both a Puppet and a Puppet Master of Sugars. Front. Plant Sci..

[78] Xiong Y., McCormack M., Li L., Hall Q., Xiang C., Sheen J. (2013). Glucose-TOR signalling reprograms the transcriptome and activates meristems. Nature.

[79] Gibson S.I. (2005). Control of plant development and gene expression by sugar signaling. Curr. Opin. Plant Biol..

[80] Borisjuk L., Walenta S., Weber H., Mueller-Klieser W., Wobus U. (2002). High-resolution histographical mapping of glucose concentrations in developing cotyledons of *Vicia faba* in relation to mitotic activity and storage processes: Glucose as a possible developmental trigger. Plant J..

[81] Sami F., Yusuf M., Faizan M., Faraz A., Hayat S. (2016). Role of sugars under abiotic stress. Plant Physiol. Biochem..

[82] Freixes S., Thibaud M.-C., Tardieu F., Muller B. (2002). Root elongation and branching is related to local hexose concentration in *Arabidopsis thaliana* seedlings. Plant Cell Environ..

[83] Jung H., Lee H.N., Marshall R.S., Lomax A.W., Yoon M.J., Kim J., Kim J.H., Vierstra R.D., Chung T. (2020). Arabidopsis cargo receptor NBR1 mediates selective autophagy of defective proteins. J. Exp. Bot..

[84] Bassham D.C. (2015). Methods for analysis of autophagy in plants. Methods.

[85] Wu Y., Yang Z., How J., Xu H., Chen L., Li K. (2017). Overexpression of a peroxidase gene (*AtPrx64*) of *Arabidopsis thaliana* in tobacco improves plant’s tolerance to aluminum stress. Plant Mol. Biol..

[86] Ma J., Liang Z., Zhao J., Wang P., Ma W., Mai K.K., Andrade J.A.F., Zeng Y., Grujic N., Jiang L. (2021). Friendly mediates membrane depolarization-induced mitophagy in *Arabidopsis*. Curr. Biol..

[87] Huang S., Taylor N.L., Ströher E., Fenske R., Millar A.H. (2013). Succinate dehydrogenase assembly factor 2 is needed for assembly and activity of mitochondrial complex II and for normal root elongation in Arabidopsis. Plant J..

[88] Sweetlove L.J., Heazlewood J.L., Herald V., Holtzapffel R., Day D.A., Leaver C.J., Millar A.H. (2002). The impact of oxidative stress on *Arabidopsis* mitochondria. Plant J..

[89] Purev M., Kim M.K., Samdan N., Yang D.-C. (2008). Isolation of a novel fructose-1,6-bisphosphate aldolase gene from *Codonopsis lanceolata* and analysis of the response of this gene to abiotic stresses. Mol. Biol..

[90] Bartel B., Farmer L.M., Rinaldi M.A., Young P.G., Danan C.H., Burkhart S.E. (2014). Mutation of the *Arabidopsis* LON2 peroxisomal protease enhances pexophagy. Autophagy.

[91] Olmedilla A., Sandalio L.M. (2019). Selective Autophagy of Peroxisomes in Plants: From Housekeeping to Development and Stress Responses. Front. Plant Sci..

[92] Park S., Gidda S.K., James C.N., Horn P.J., Khuu N., Seay D.C., Keereetaweep J., Chapman K.D., Mullen R.T., Dyer J.M. (2013). The α/β hydrolase CGI-58 and peroxisomal transport protein PXA1 coregulate lipid homeostasis and signaling in *Arabidopsis*. Plant Cell.

[93] Sanchez Carranza A.P., Singh A., Steinberger K., Panigrahi K., Palme K., Dovzhenko A., Dal Bosco C. (2016). Hydrolases of the ILR1-like family of *Arabidopsis thaliana* modulate auxin response by regulating auxin homeostasis in the endoplasmic reticulum. Sci. Rep..

[94] Elena F., Feraru I.M., Elke B., Sascha W., Lin S., Angelika G., Kleine-Vehn J. (2019). PILS6 is a temperature-sensitive regulator of nuclear auxin input and organ growth in *Arabidopsis thaliana*. Proc. Natl. Acad. Sci. USA.

[95] Hofius D., Schultz-Larsen T., Joensen J., Tsitsigiannis D.I., Petersen N.H.T., Mattsson O., Jørgensen L.B., Jones J.D.G., Mundy J., Petersen M. (2009). Autophagic Components Contribute to Hypersensitive Cell Death in *Arabidopsis*. Cell.

[96] Honig A., Avin-Wittenberg T., Ufaz S., Galili G. (2012). A new type of compartment, defined by plant-specific Atg8-interacting proteins, is induced upon exposure of *Arabidopsis* plants to carbon starvation. Plant Cell.

[97] Schneider C.A., Rasband W.S., Eliceiri K.W. (2012). NIH Image to ImageJ: 25 years of image analysis. Nat. Methods.

[98] Di Berardino J., Marmagne A., Berger A., Yoshimoto K., Cueff G., Chardon F., Masclaux-Daubresse C., Reisdorf-Cren M. (2018). Autophagy controls resource allocation and protein storage accumulation in Arabidopsis seeds. J. Exp. Bot..

[99] Fling S.P., Gregerson D.S. (1986). Peptide and protein molecular weight determination by electrophoresis using a high-molarity tris buffer system without urea. Anal. Biochem..

[100] Towbin H., Staehelint T., Gordon J. (1979). Electrophoretic transfer of proteins from polyacrylamide gels to nitrocellulose sheets: Procedure and some applications. Proc. Natl. Acad. Sci. USA.

[101] Yu T.-S., Kofler H., Häusler R.E., Hille D., Flügge U.-I., Zeeman S.C., Smith A.M., Kossmann J., Lloyd J., Ritte G. (2001). The Arabidopsis *sex1* Mutant Is Defective in the R1 Protein, a General Regulator of Starch Degradation in Plants, and Not in the Chloroplast Hexose Transporter. Plant Cell.

[102] Kötting O., Pusch K., Tiessen A., Geigenberger P., Steup M., Ritte G. (2005). Identification of a novel enzyme required for starch metabolism in arabidopsis leaves. The phosphoglucan, water dikinase. Plant Physiol..

[103] Lisec J., Schauer N., Kopka J., Willmitzer L., Fernie A.R. (2006). Gas chromatography mass spectrometry—Based metabolite profiling in plants. Nat. Protoc..

[104] Dhatt B.K., Abshire N., Paul P., Hasanthika K., Sandhu J., Zhang Q., Obata T., Walia H. (2019). Metabolic Dynamics of Developing Rice Seeds Under High Night-Time Temperature Stress. Front. Plant Sci..

[105] Rödiger A., Agne B., Baerenfaller K., Baginsky S. (2014). Arabidopsis Proteomics: A Simple and Standardizable Workflow for Quantitative Proteome Characterization. Methods Mol. Biol..

[106] Wessel D., Flügge U.I. (1984). A method for the quantitative recovery of protein in dilute solution in the presence of detergents and lipids. Anal. Biochem..

[107] Scheltema R.A., Hauschild J.P., Lange O., Hornburg D., Denisov E., Damoc E., Kuehn A., Makarov A., Mann M. (2014). The Q exactive HF, a benchtop mass spectrometer with a pre-filter, high-performance quadrupole and an ultra-high-field orbitrap analyzer. Mol. Cell. Proteom..

[108] Rgen Cox J., Hein M.Y., Luber C.A., Paron I., Nagaraj N., Mann M. (2014). Accurate Proteome-wide Label-free Quantification by Delayed Normalization and Maximal Peptide Ratio Extraction, Termed MaxLFQ. Mol. Cell. Proteom..

[109] Tyanova S., Temu T., Sinitcyn P., Carlson A., Hein M.Y., Geiger T., Mann M., Cox J. (2016). The Perseus computational platform for comprehensive analysis of (prote)omics data. Nat. Methods.

[110] RStudio Team (2020). RStudio: Integrated Development for R. RStudio.

[111] Wickham H., Bryan J. (2019). Readxl: Read Excel Files. R Package Version 1.3.1. https://CRAN.R-project.org/package=readxl.

[112] Vincent Q.V. (2011). Ggbiplot: A Ggplot2 Based Biplot. R Package Version 0.55. http://github.com/vqv/ggbiplot.

[113] Wickham H. (2016). Ggplot2: Elegant Graphics for Data Analysis.

[114] Slowikowski K. (2021). Ggrepel: Automatically Position Non-Overlapping Text Labels with ‘Ggplot2’. R Package Version 0.9.1. https://CRAN.R-project.org/package=ggrepel.

[115] Huang D.W., Sherman B.T., Lempicki R.A. (2009). Systematic and integrative analysis of large gene lists using DAVID bioinformatics resources. Nat. Protoc..

[116] Howe E., Holton K., Nair S., Schlauch D., Sinha R., Quackenbush J. (2010). MeV: MultiExperiment Viewer. Biomedical Informatics for Cancer Research.

