# Supplementary material for: Exploring the Contribution of Autophagy to the Excess-Sucrose Response in Arabidopsis thaliana
Source: Int J Mol Sci. 2022 Mar 31;23(7):3891. doi: 10.3390/ijms23073891 (PMC8999498; doi:10.3390/ijms23073891)
Supplement: Supplementary file 1 [file ijms-23-03891-s001.zip › ijms-1552964-supplementary .pdf]

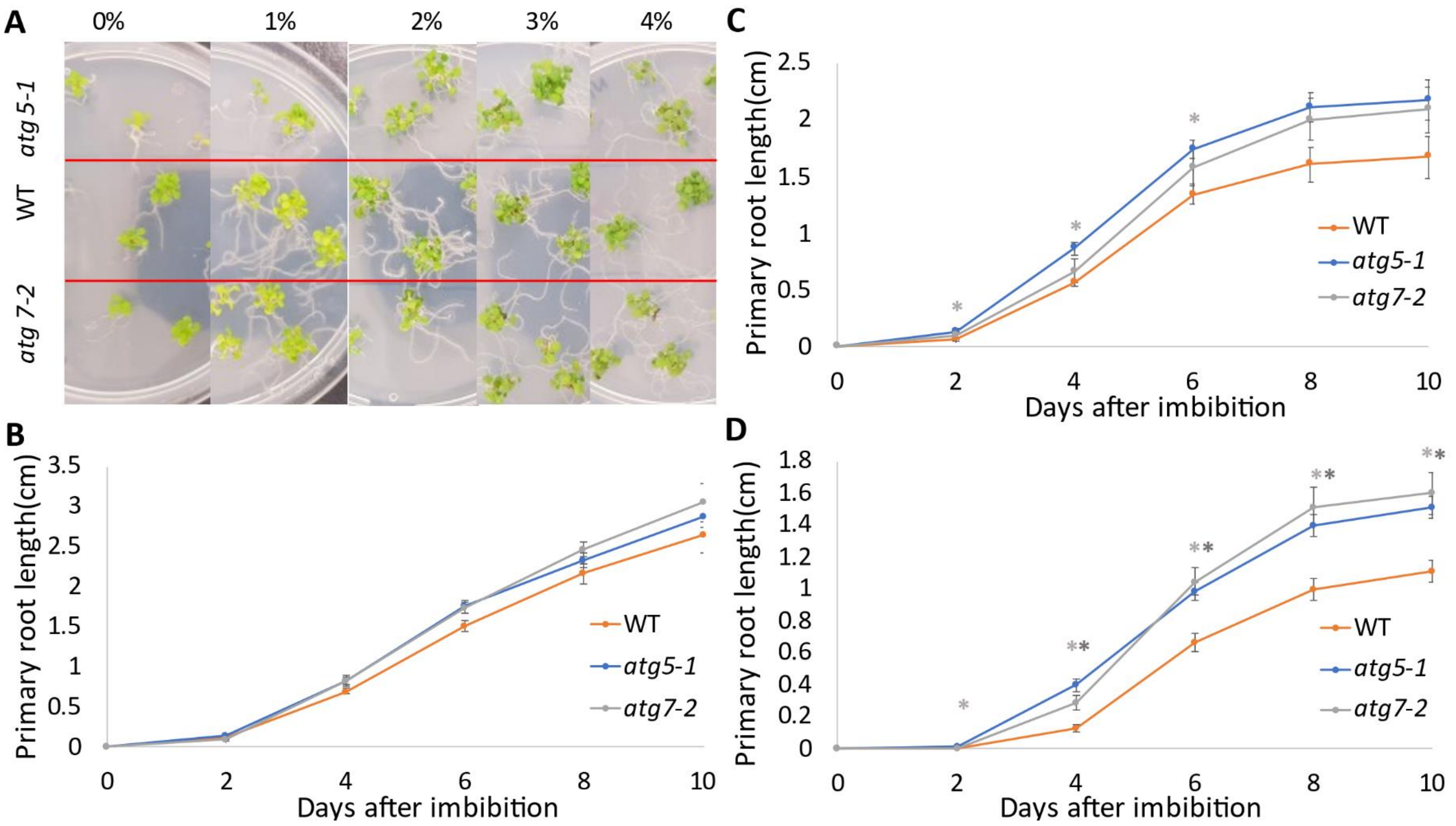

Figure S1: root elongation is less inhibited by sucrose excess in *atg* mutants. (A) WT, *atg*5-1, and *atg*7-2 plants were sown on Nitsch plates contacting 0-4% sucrose and grown for 14 days. Representative images of the seedlings under various sucrose concentrations. (B-D) WT, *atg*5-1 and *atg*7-2 plants were sown on Nitsch plates contacting 1% (B) 2% (C) or 3% (D) sucrose. Primary root length was measured every two days for 10 days after imbibition. Data are presented as average ± SE. Asterisk denotes a significant difference from WT at the same treatment and time point by Dunnett's test ($p<0.05$, n=8-15), light grey – *atg5-1*, dark grey – *atg7-2*.

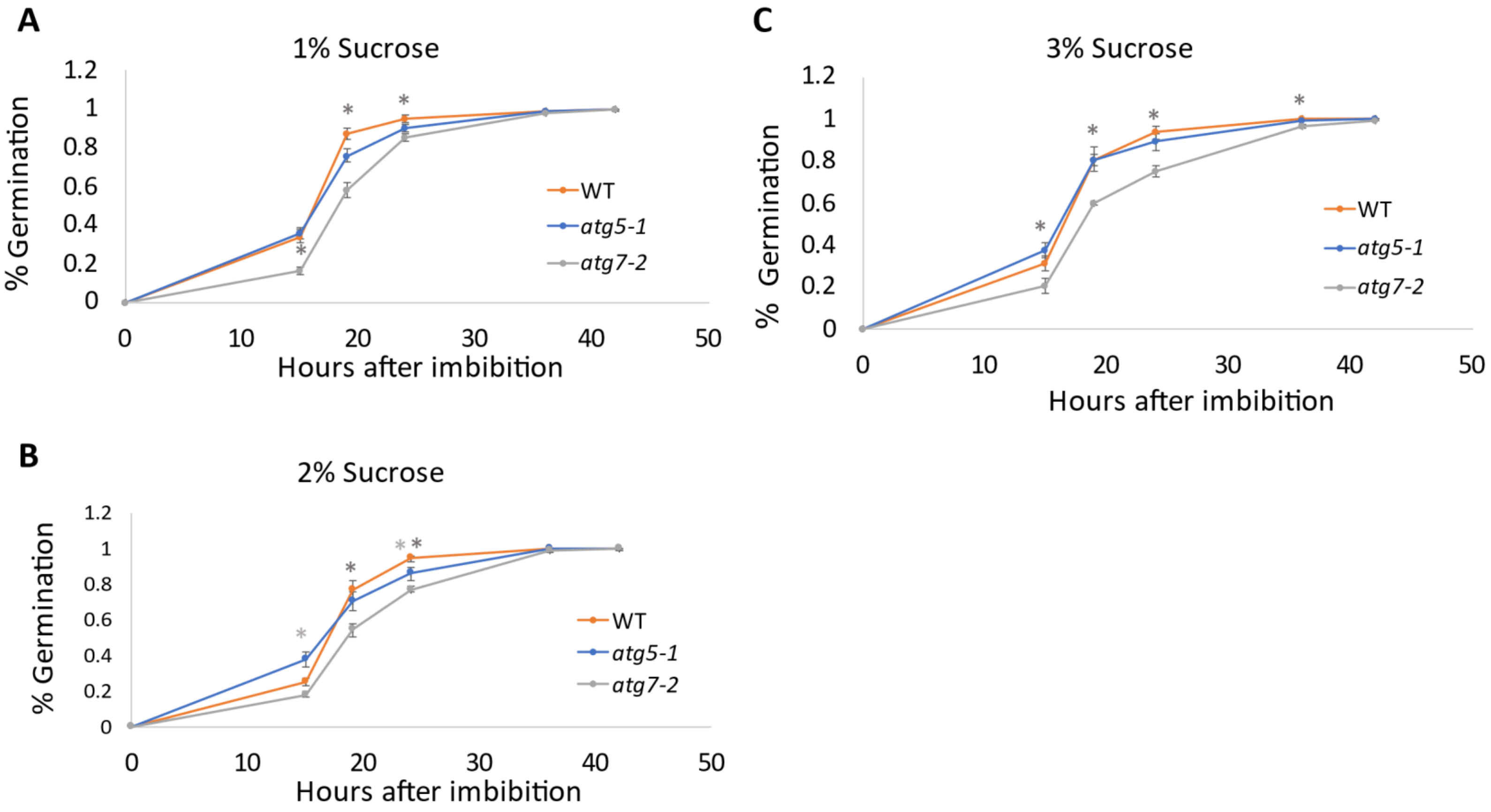

Figure S2: Germination rate is slightly higher in WT plants under sucrose treatments. WT, *atg5-1* and *atg7-2* plants were sown on Nitsch medium plates contacting 1% (A) 2% (B) or 3% (C) sucrose. GP was calculated by observing how many seeds germinated/total seeds. Seeds were defined as germinated when radicle protrusion was observed. Data are presented as average ± SE. Asterisk denotes a significant difference from WT at the same treatment and time point by Dunnett's test (p<0.05, n=4-6), light grey – *atg5-1*, dark grey – *atg7-2*.

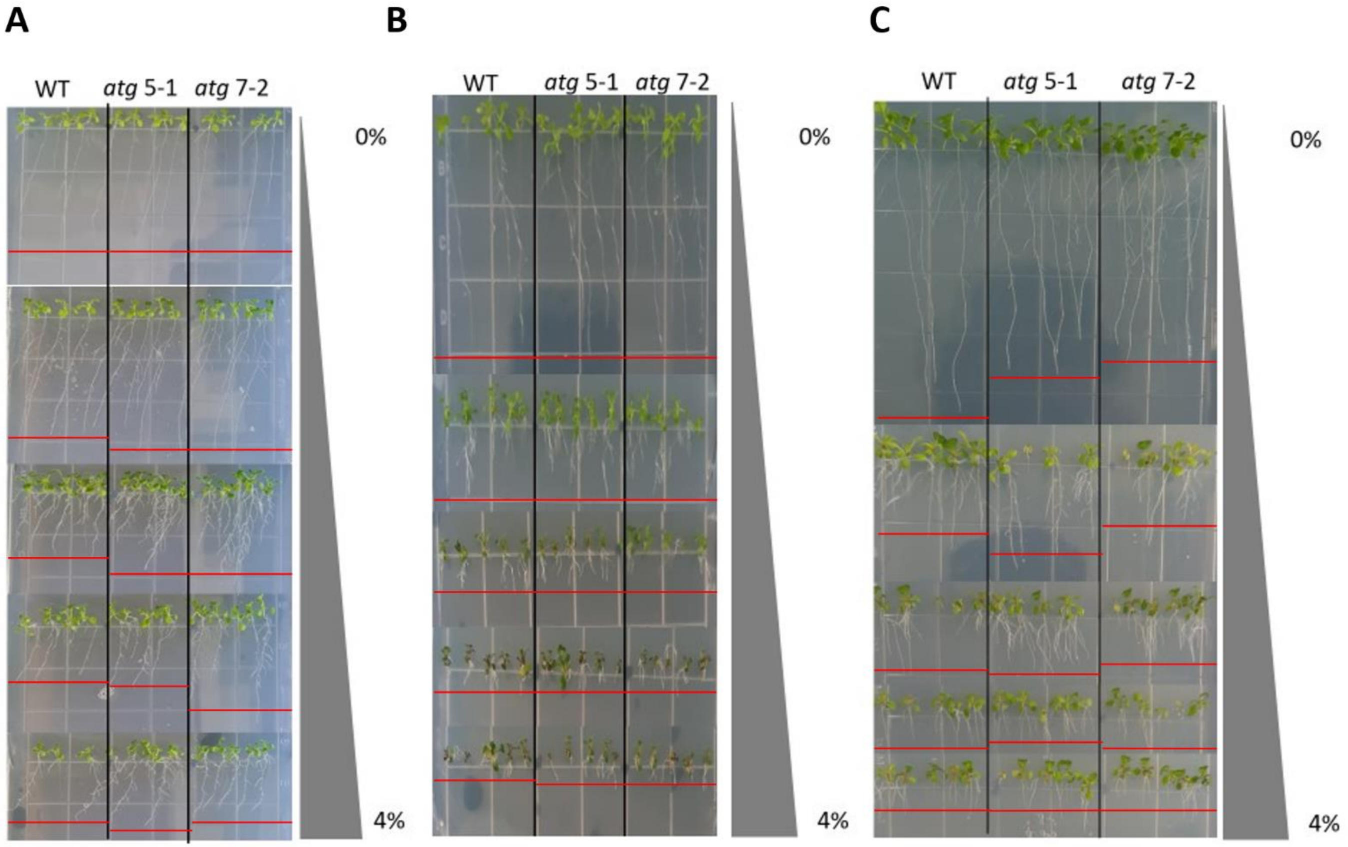

Figure S3: Reduced root length inhibition in *atg* mutants occurs only under sucrose and glucose excess. WT, *atg5-1,* and *atg7-2* plants were sown on Nitsch plates containing increasing concentrations of Glucose (A), Fructose (B), and Mannitol (C). The plants were grown vertically for 14 days. Pictures display representative images of the plants.

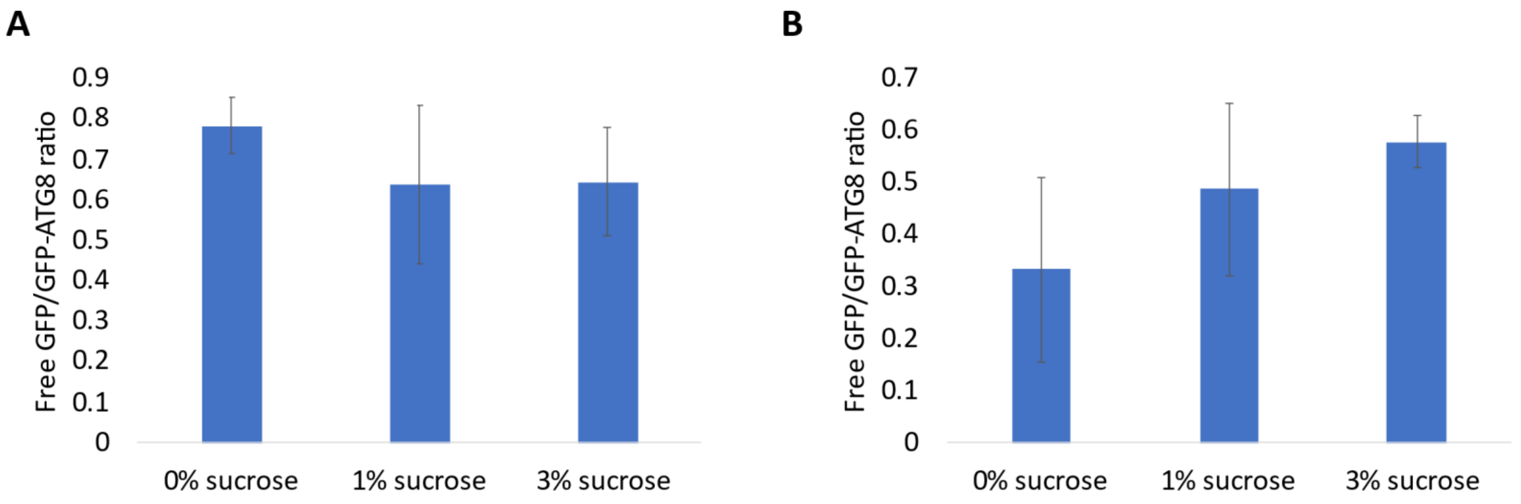

Figure S4: GFP release assay shows basal autophagic activity in the roots and induced autophagy in the shoots under sucrose excess. The ratio between the pixel area of Free GFP and GFP-ATG8 bands in the roots (A) and shoots (B) as determined by ImageJ analysis of Western blots. Data are presented as average ± SE. No significant difference was observed by Dunnett's test (p<0.05, n=3). However, a clear trend was observed in shoots under increasing sucrose concentrations.

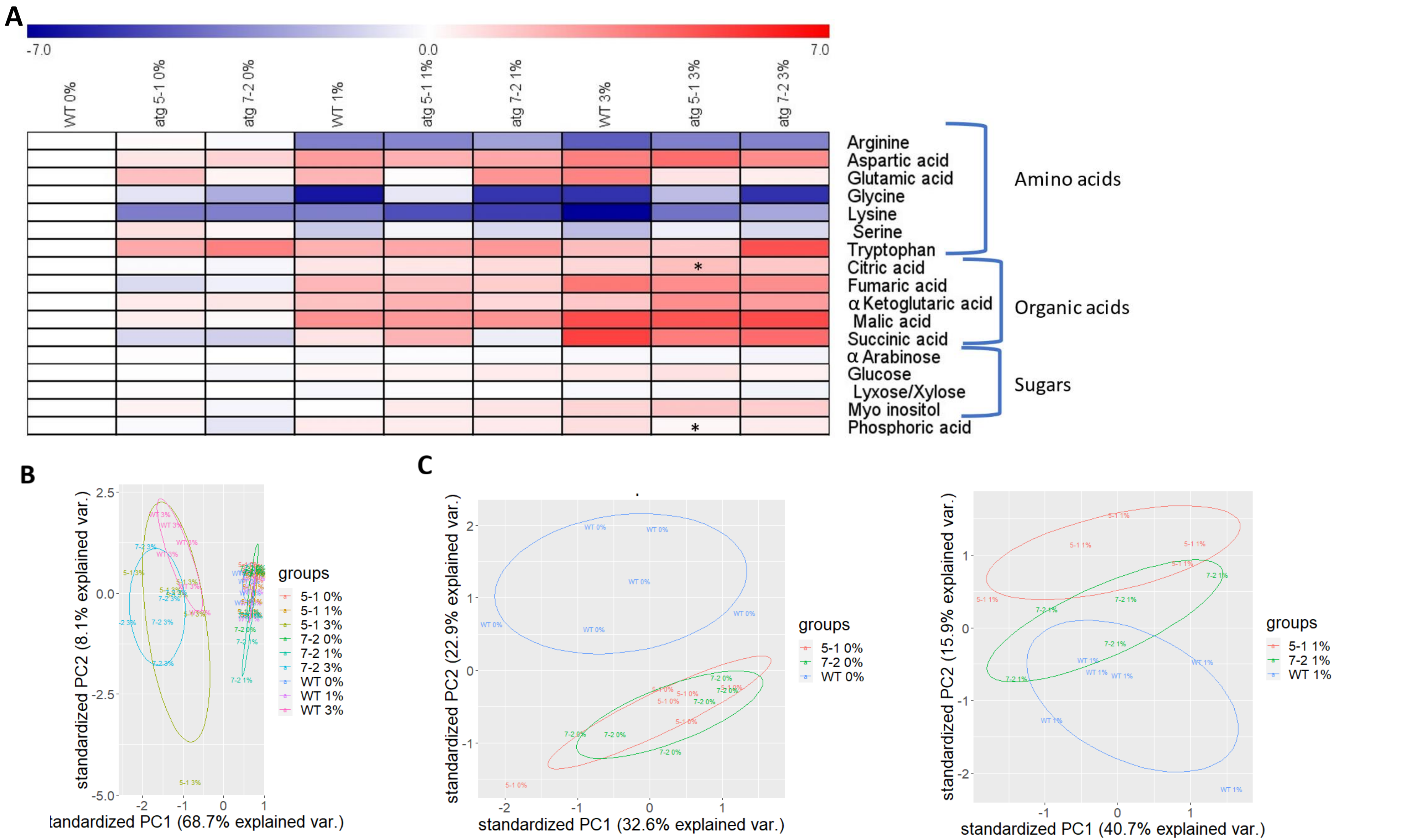

Figure S5: Sucrose excess affects shoot metabolome in an autophagy-independent manner. 10-day-old roots and shoots from WT, *atg5-1,* and *atg7-2* seedlings grown under various sucrose concentrations were collected separately, and polar metabolites were extracted and analyzed by GC-MS (A) Heatmap describing the relative amounts of shoot metabolites under various sucrose concentrations. Results are presented as Log2 ratio normalized to WT 0%. Asterisk denotes a significant difference from WT at the same treatment by Dunnett's test ($p<0.05$, n=4-6). (B-D) PCA of root metabolites under 0% sucrose (B), 1% sucrose (C), and all treatments combined (D).

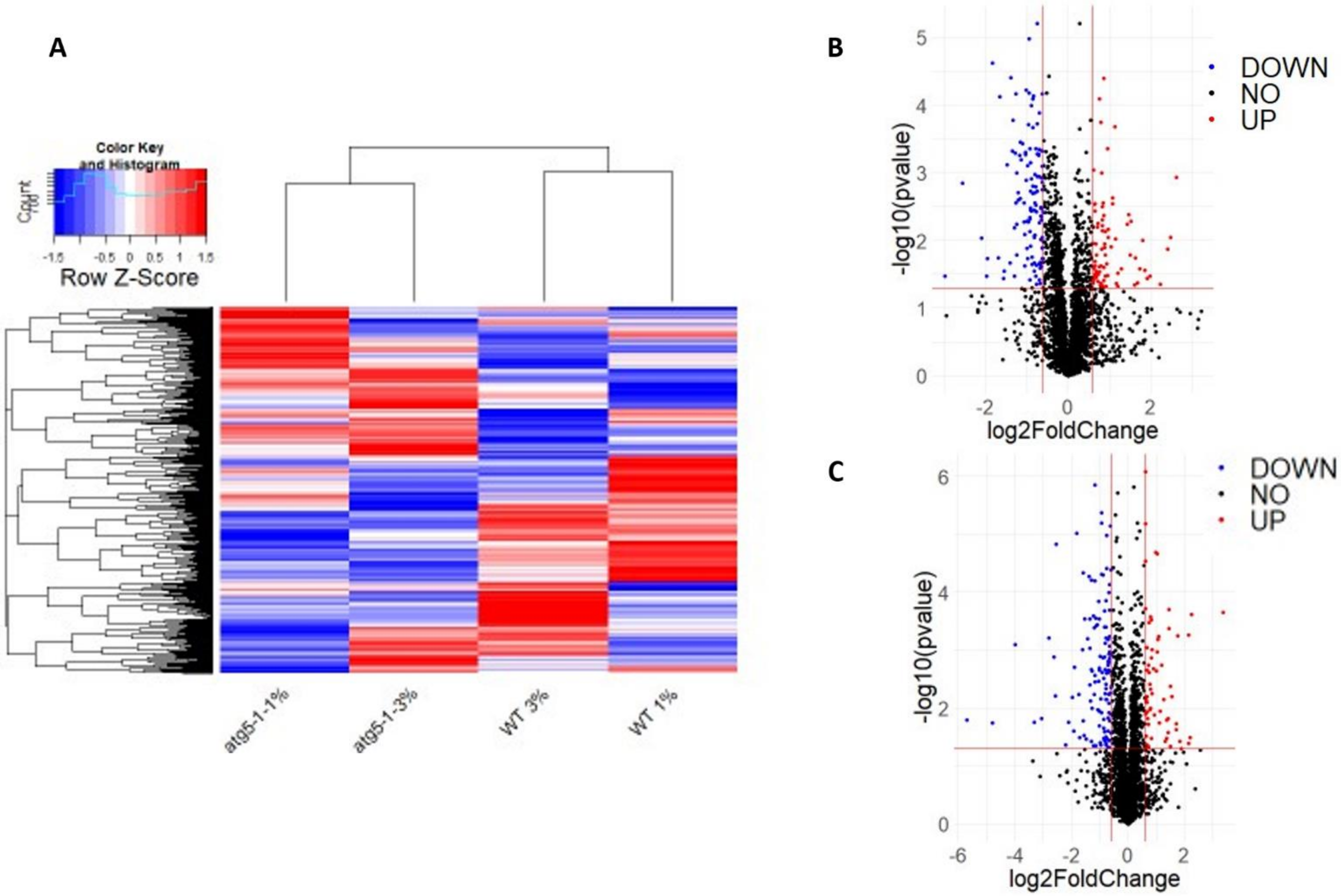

Figure S6: Proteomic analysis of roots reveals differences in protein levels during sucrose excess and between WT and *atg5* plants. (A) Heatmap of the entire proteomic profile. (B) Volcano plot for WT proteome in 1% and 3% sucrose- *p*-values and fold change relative to WT 3%. (C) Volcano plot for *atg5-1* plants in 1% and 3% sucrose – *p*-values and fold change relative to *atg5-1* 3%.

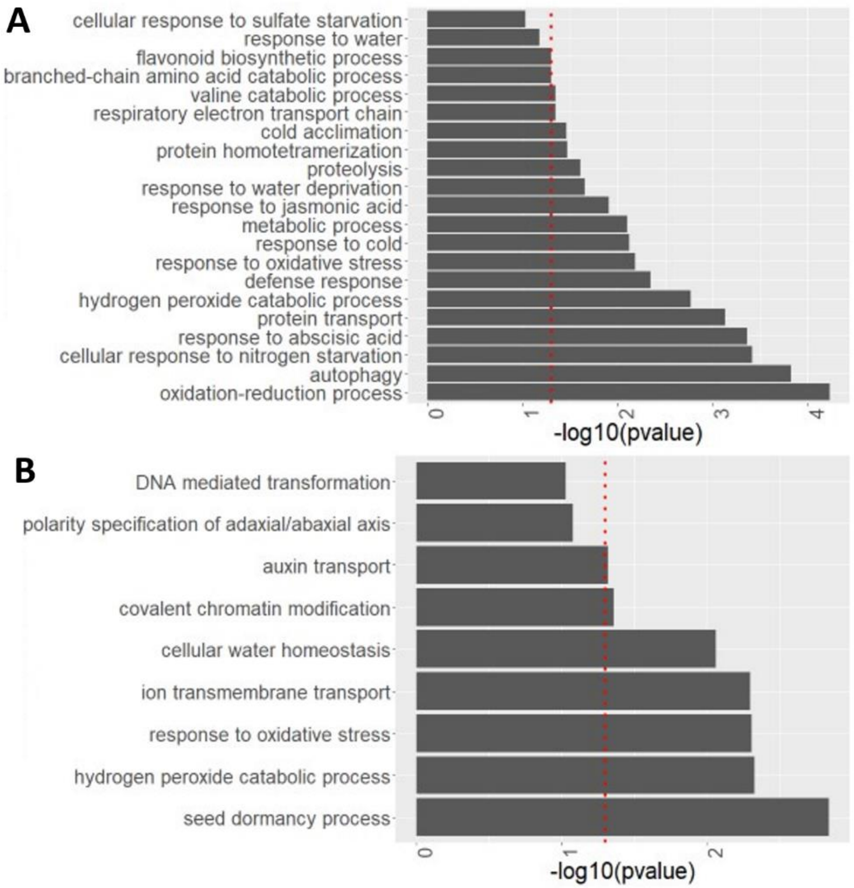

Figure S7: Biological processes differences under 1% sucrose. Enriched biological processes arising from the proteomics were identified using David functional annotation tools, using GOTERM_BP_DIRECT. (A) Biological processes enriched for proteins that accumulated in *atg5-1* plants under 1% sucrose. (B) Biological processes enriched for proteins that decreased in *atg5-1* plants under 1% sucrose. The red dashed line indicates the threshold for significant enrichment of the given proteins for the biological process(P<0.05).

Table S1. Shoot metabolites. Metabolic profiling of 10-day-old seedling shoots. Seedling shoots were collected from mediums containing various concentrations of sucrose. Values represent average ± standard error; values are all normalized to average WT 0%. Significant differences calculated by Dunnett's test (*p*<0.05, n=4-6) compared with WT under the same treatment are highlighted in bold

Group	Metabolite	WT 0%	*atg* 5-1 0%	*atg* 7-2 0%	WT 1%	*atg* 5-1 1%	*atg* 7-2 1%	WT 3%	*atg* 5-1 3%	*atg* 7-2 3%
	Aspartic acid	1±0.66	1.63±0.61	2.30±0.51	6.57±0.67	4.46±0.97	5.18±0.93	11.25±1.41	15.83±1.13	8.59±2.19
	Arginine	1±0.19	1.10±0.33	0.91±0.26	0.09±0.06	0.10±0.03	0.17±0.03	0.05±0.02	0.09±0.03	0.10±0.06
	Glutamic acid	1±0.10	3.28±2.21	1.22±0.02	4.12±2.98	1.07±0.16	7.58±4.04	10.43±4.32	1.65±0.16	1.35±0.18
Amino acids	Glycine	1±0.46	0.61±0.41	0.21±0.16	0.01±0.005	0.64±0.52	0.02±0.009	0.02±0.01	0.28±0.14	0.02±0.009
	Lysine	1±0.63	0.09±0.02	0.09±0.02	0.09±0.08	0.04±0.02	0.03±0.01	0.008±0.004	0.07±0.03	0.19±0.10
	Serine	1±0.50	1.83±0.61	1.19±0.39	0.37±0.13	0.81±0.38	0.49±0.12	0.28±0.09	0.75±0.17	0.49±0.13
	Tryptophan	1±0.99	5.10±2.80	10.82±8.62	4.27±2.90	5.18±2.50	6.98±5.21	3.45±1.91	2.83±1.34	27.35±19.43
	Citric acid	1±0.13	0.93±0.08	0.85±0.04	1.62±0.10	1.58±0.24	1.98±0.08	2.13±0.23	**3.41±0.31**	2.81±0.40
	Fumaric acid	1±0.13	0.50±0.11	0.75±0.24	3.98±0.49	3.29±1.69	2.58±0.60	13.09±1.67	8.81±1.78	8.59±1.70
Organic acids	Alpha ketoglutaric acid	1±0.52	1.48±0.59	1.63±0.69	3.25±1.07	4.46±0.89	2.09±0.98	2.81±1.04	8.73±2.39	6.51±2.68
	Malic acid	1±0.31	1.40±0.61	1.10±0.45	7.92±0.43	6.99±2.58	7.67±1.38	30.08±4.44	26.08±4.69	31.12±8.51
	Succinic acid	1±0.30	0.48±0.18	0.41±0.12	1.67±1.01	4.19±3.43	0.73±0.09	37.18±7.69	11.87±8.39	16.24±9.32
	Alpha arabinose	1±0.10	1.07±0.10	1.01±0.10	0.89±0.05	0.95±0.09	0.94±0.04	0.90±0.09	0.90±0.01	0.81±0.07
	Glucose	1±0.10	1.02±0.05	0.87±0.06	1.22±0.04	1.25±0.16	1.47±0.05	1.60±0.12	1.74±0.10	1.31±0.35
	Lyxose/Xylose	1±0.10	1.06±0.10	1±0.10	0.88±0.05	0.95±0.08	0.97±0.04	0.90±0.09	0.89±0.02	0.81±0.07
Sugars	Myo inositol	1±0.18	1.28±0.15	0.83±0.12	1.03±0.07	1.50±0.36	1.68±0.19	2.20±0.32	2.99±0.10	2.49±0.20
Others	Phosphoric acid	1±0.15	0.91±0.13	0.61±0.08	1.47±0.11	1.42±0.28	1.51±0.19	1.89±0.17	**1.17±0.20**	1.45±0.07

Table S2. Root metabolites. Metabolic profiling of 10-day-old seedling roots. Seedling roots were collected from mediums containing various concentrations of sucrose. Values represent average ± standard error; values are all normalized to average WT 0%. Significant differences calculated by Dunnett's test (*p*<0.05 n=4-6) compared with WT under the same treatment are highlighted in bold

**Group**	**Metabolite**	**WT 0%**	***atg* 5-1 0%**	***atg* 7-2 0%**	**WT 1%**	***atg* 5-1 1%**	***atg* 7-2 1%**
Amino acids	Aspartic acid	1±0.31	0.25±0.11	0.75±0.50	1.5±0.25	0.85±0.22	1.92±0.62
	Arginine	1±0.24	**0.28±0.08**	**0.12±0.06**	0.006±0.002	0.003±0.002	0.005±0.002
	Glutamic acid	1±0.11	0.66±0.04	0.94±0.26	0.88±0.15	0.76±0.08	1.16±0.21
	Glycine	1±0.32	**0.25±0.04**	**0.17±0.02**	0.14±0.02	0.10±0.009	0.10±0.01
	Methionine	1±0.22	0.75±0.10	0.78±0.20	1.15±0.33	1.25±0.24	2.13±0.36
	Ornithine	1±0.30	0.61±0.29	0.25±0.19	1.16±0.27	1.78±0.29	1.75±0.28
	Phenylalanine	1±0.35	**0.07±0.04**	**0.08±0.06**	0.02±0.01	0.03±0.02	0.02±0.01
	Proline	1±0.38	0.23±0.11	0.21±0.11	0.12±0.06	0.06±0.04	0.09±0.04
	Serine	1±0.18	**0.51±0.13**	**0.40±0.07**	0.14±0.01	0.14±0.02	0.13±0.02
	Threonine	1±0.21	**0.11±0.03**	**0.14±0.02**	0.39±0.05	**0.21±0.03**	0.29±0.06
	Tyrosine	1±0.14	0.48±0.04	0.68±0.33	1.46±0.42	2.10±0.63	**4.48±1.15**
Organic acids	Citric acid	1±0.26	1.10±0.28	1.64±0.41	9.28±1.48	12.77±1.46	13.03±1.58
	Fumaric acid	1±0.32	0.60±0.18	0.58±0.10	0.76±0.06	0.53±0.08	0.78±0.16
	Alpha ketoglutaric acid	1±0.47	1.11±0.42	1.45±0.32	2.08±0.20	3.05±0.57	2.65±0.41
	Malic acid	1±0.22	1.87±0.42	2.34±0.59	6.19±0.66	4.45±0.66	5.80±0.64
	Succinic acid	1±0.20	1.20±0.34	1.10±0.21	2.61±0.32	1.85±0.38	2.05±0.23
Sugars	Alpha arabinose	1±0.11	1.12±0.14	0.90±0.09	0.46±0.04	0.47±0.07	0.49±0.05
	Fructose	1±0.30	1.88±0.57	0.80±0.37	12.08±2.63	18.62±2.72	19.29±2.95
	Glucose	1±0.16	1.26±0.09	1.23±0.05	0.38±0.18	0.12±0.02	0.13±0.02
	Lyxose/Xylose	1±0.07	1.10±0.09	0.92±0.07	0.47±0.03	0.46±0.06	0.49±0.04
	Myo inositol	1±0.26	1.27±0.24	1.29±0.20	2.52±0.14	2.85±0.25	2.78±0.19
	Sucrose	1±1	**8.24±2.67**	6.39±1.58	10.75±2.63	**30.73±4.20**	13.73±1.53
	Trehalose	1±1	**7.02±2.47**	3.72±1.13	2.21±1.46	0.10±0.10	1.70±0.32
others	Phosphoric acid	1±0.07	0.83±0.08	**0.71±0.03**	0.46±0.04	0.39±0.05	0.41±0.04
